# MicrobiomeGWAS: A Tool for Identifying Host Genetic Variants Associated with Microbiome Composition

**DOI:** 10.3390/genes13071224

**Published:** 2022-07-09

**Authors:** Xing Hua, Lei Song, Guoqin Yu, Emily Vogtmann, James J. Goedert, Christian C. Abnet, Maria Teresa Landi, Jianxin Shi

**Affiliations:** 1 Division of Cancer Epidemiology and Genetics, National Cancer Institute, National Institute of Health, Rockville, MD 20850, USA; xhua2@fredhutch.org (X.H.); lei.song@nih.gov (L.S.); emily.vogtmann@nih.gov (E.V.); jamesjgoedert@gmail.com (J.J.G.); abnetc@mail.nih.gov (C.C.A.); landim@mail.nih.gov (M.T.L.); 2 Public Health Sciences Division, Fred Hutchinson Cancer Research Center, Seattle, WA 98109, USA; 3 Molecular Genetics and Genomics Branch, Center for Scientific Review, National Institute of Health, Bethesda, MD 20817, USA; guoqin.yu@nih.gov

**Keywords:** microbiome, genome-wide association study, gene–environment interaction, host genetics, tail probabilities, skewness and kurtosis

## Abstract

The microbiome is the collection of all microbial genes and can be investigated by sequencing highly variable regions of 16S ribosomal RNA (rRNA) genes. Evidence suggests that environmental factors and host genetics may interact to impact human microbiome composition. Identifying host genetic variants associated with human microbiome composition not only provides clues for characterizing microbiome variation but also helps to elucidate biological mechanisms of genetic associations, prioritize genetic variants, and improve genetic risk prediction. Since a microbiota functions as a community, it is best characterized by β diversity; that is, a pairwise distance matrix. We develop a statistical framework and a computationally efficient software package, microbiomeGWAS, for identifying host genetic variants associated with microbiome β diversity with or without interacting with an environmental factor. We show that the score statistics have positive skewness and kurtosis due to the dependent nature of the pairwise data, which makes *p*-value approximations based on asymptotic distributions unacceptably liberal. By correcting for skewness and kurtosis, we develop accurate *p*-value approximations, whose accuracy was verified by extensive simulations. We exemplify our methods by analyzing a set of 147 genotyped subjects with 16S rRNA microbiome profiles from non-malignant lung tissues. Correcting for skewness and kurtosis eliminated the dramatic deviation in the quantile–quantile plots. We provided preliminary evidence that six established lung cancer risk SNPs were collectively associated with microbiome composition for both unweighted (*p* = 0.0032) and weighted (*p* = 0.011) UniFrac distance matrices. In summary, our methods will facilitate analyzing large-scale genome-wide association studies of the human microbiome.

## 1. Introduction

The human body is colonized by bacteria, viruses, and other microbes that exceed the number of human cells by at least 10-fold and that exceed the number of human genes by at least 100-fold. The relationship between a person and his or her microbial population, termed the microbiota, is generally mutualistic. The microbiota may promote human health by inhibiting infection by pathogens, conditioning the immune system, synthesizing and digesting nutrients, and maintaining overall homeostasis. The microbiome, which is the collection of all microbial genes, can be investigated through massively parallel, next-generation DNA sequencing technologies. By amplifying and sequencing highly variable regions of 16S ribosomal RNA genes that are present in all eubacteria, cost-effective and informative microbiome profiles down to the genus level are obtained.

The human microbiome has been associated with diseases, including obesity [1], inflammatory bowel disease (IBD) [2], colorectal cancer [3], and breast cancer [4]. Thus, identifying factors that have a sustained impact on the microbiome is fundamental for elucidating its role in health conditions and for developing treatment strategies. Increasing evidence suggests that microbiome composition at a specific site of the human body is impacted by environmental factors [5,6], host genetics [7,8], and possibly by their interactions. In the mouse, quantitative trait loci (QTL) studies have identified loci contributing to the variation in the gut microbiome using linkage analysis [9,10]. Recently, Goodrich et al. [11] systematically investigated the heritability of the human gut microbiome by comparing monozygotic twins to dizygotic twins and found substantial heritability in different microbiome metrics, suggesting the important role of host genetics on gut microbiome diversity. Associations between individual host genetic variants and microbiome taxa abundances have also begun to emerge in other human samples [7,8,12]. These studies suggest that genome-wide association studies (GWAS) have great potential to identify host genetic variants associated with microbiome diversity.

GWAS of complex human diseases have identified many risk SNPs; however, the biological mechanisms are largely unknown for the majority of the risk SNPs. QTL studies of intermediate traits, e.g., gene expression [13,14], DNA methylation [15,16], chromatin structure [17,18], and metabolite production [19,20], have provided useful insights into the biological mechanisms of the GWAS findings. The human microbiome at a specific body site is another important and informative intermediate trait for interpreting GWAS signals. Knights et al. [8] reported that a risk SNP for IBD located in *NOD2* was associated with the relative abundance of *Enterobacteriaceae* in the human gut microbiome. Tong et al. [7] show that a loss-of-function allele in *FUT2* that increases the risk of developing Crohn’s Disease (CD) may modulate the energy metabolism of the gut microbiome. In both examples, the microbiome is a potential intermediate for explaining the association between risk SNPs and disease risks, although a formal mediation analysis is required based on samples with genotype, microbiome, and disease status data. Moreover, identifying microbiome-associated host genetic variants has the potential to prioritize SNPs for discovery and to improve the performance of polygenetic risk prediction.

Three types of microbiome metrics can be derived as phenotypes for GWAS analysis. First, for each taxon at a specified taxonomic level (phylum, class, order, family, genus, and species), we calculate the relative abundance (RA) of the taxon as the ratio of the number of sequencing reads assigned to the taxon to the total number of sequencing reads. In 16S ribosomal RNA sequence profiles, approximately 100–200 taxa with average RAs ≥ 0.1% (from the phylum level to the genus level) across samples are abundant enough for QTL analysis. One can perform a Poisson regression to examine the association between the RA of each taxon and each SNP. Significant associations are identified using Bonferroni correction (*p* < 5 × 10^−8^/200 = 2.5 × 10^−10^) or by controlling FDR at an appropriate level. Second, multiple α-diversity metrics [21] can be calculated to reflect the richness (e.g., number of unique taxa) and evenness of each microbiome community after a procedure called rarefication, which eliminates the dependence between the estimated α diversity and the variable total number of sequence reads across subjects. Once the α-diversity metrics are derived, one may perform standard GWAS with α diversity as the phenotype using linear regression.

Because a microbiota functions as a community, the most important analysis for a microbiome GWAS may be by assessing the complete structure of the community by using a pairwise microbiome distance matrix (or β diversity) of the microbial community. Microbiome distances can be defined in different ways, based on using phylogenetic tree information or each taxon’s abundance information. Bray–Curtis dissimilarity [22] quantifies the difference between two microbiome communities using the abundance information of specific taxa. UniFrac [23,24,25] is another widely used distance metric. Unlike the Bray–Curtis dissimilarity metric, UniFrac compares microbiome communities by using information on the relative relatedness of each taxon, specifically by phylogenetic distance (branch lengths on a phylogenetic tree). UniFrac has two variants: the weighted UniFrac [24], which accounts for the taxa abundance information, and the unweighted UniFrac [23], which only models the information of presence or absence. Recently, a generalized UniFrac distance metric [26] was developed to automatically appreciate the advantages of weighted and unweighted UniFrac metrics and was shown to provide better statistical power to detect associations between human health conditions and microbiome communities. GWAS based on a microbiome distance matrix aims to identify the host SNPs associated with microbiome composition. This has been done frequently by fitting non-parametric multivariate models [27]. This approach requires permutations to assess significance [28], which is computationally prohibitive, particularly when evaluating *p*-values less than 5 × 10^−8^—the standard GWAS *p*-value threshold—or even lower when testing multiple-diversity matrices. In a recent microbiome GWAS, the computation is prohibitive even using a moment matching method based on the F-statistic.

Intuitively, the microbiome distances tend to be smaller for pairs of subjects with similar genotypic values at the associated SNP. In addition, it is also of great interest to identify host SNPs that interact with an environmental factor to affect microbiome composition. Importantly, β diversity is temporally more stable compared with RA of taxa and α-diversity metrics based on the data from the Human Microbiome Project [29], suggesting a smaller power loss for a GWAS due to temporal variability. To our knowledge, no statistical methods or software packages have been designed to efficiently analyze microbiome GWAS data using distance matrices as phenotypes.

In this paper, we develop a statistical framework and a computationally efficient package, microbiomeGWAS, for analyzing microbiome GWAS data. Our package allows the detection of host SNPs with the main effect or interaction with an environment factor; i.e., host SNPs interacting with an environment factor to affect the microbiome composition. We calculate the variance of the score statistics by appropriately considering the dependence of the pairwise distances. Importantly, we show that the score statistics have positive skewness and kurtosis due to the dependence in pairwise distances, which makes the approximation of small *p*-values based on the asymptotic distribution too liberal, which easily yields false positive associations. Resampling methods, e.g., bootstrap or permutation, are computationally prohibitive for accurately approximating small *p*-values. We propose to improve the tail probability approximation by correcting for skewness and kurtosis of the score statistics. Numerical investigations demonstrate that our method provides a very accurate approximation, even for *p* = 5 × 10^−8^. MicrobiomeGWAS runs very efficiently, taking 36 min for analyzing main effects and 69 min for analyzing both main and interaction effects for a study with 2000 subjects and 500,000 SNPs, using a single core. MicrobiomeGWAS is available at https://github.com/lsncibb/microbiomeGWAS [30], accessed on 30 May 2022.

We illustrate our methods by applying microbiomeGWAS to non-malignant lung tissue samples (N=147) in the Environment And Genetics in Lung cancer Etiology (EAGLE) study [31,32]. Because smoking may alter microbiome composition, we tested both the main effect and gene–smoking interaction effect. When *p*-values were calculated based on asymptotic distributions, the quantile–quantile (QQ) plots strongly deviated from the uniform distribution. Nine loci also achieved genome-wide significance based on asymptotic approximations. Correcting for skewness and kurtosis eliminated the inflation and also the genome-wide significance of these loci. However, we provide evidence that the established lung cancer risk SNPs are associated with lung microbiome composition.

## 2. Material and Methods

### 2.1. A Score Statistic for Testing Main Effect

Suppose that we have a set of N subjects genotyped with SNP arrays. For notational simplicity, we consider only one SNP with a minor allele frequency (MAF) denoted as f. Our interest centers on testing whether the genotype of the SNP is associated with microbiome composition. Let $g_{n}=0,1,2$ represent the number of the minor alleles for subject n. We assume that the 16S rRNA gene of microbiota from a target site (e.g., gut) has been sequenced for these samples. Let $d_{ij}$ be the microbiome distance between subject i and subject j and D be the distance matrix.

**Figure 1:**
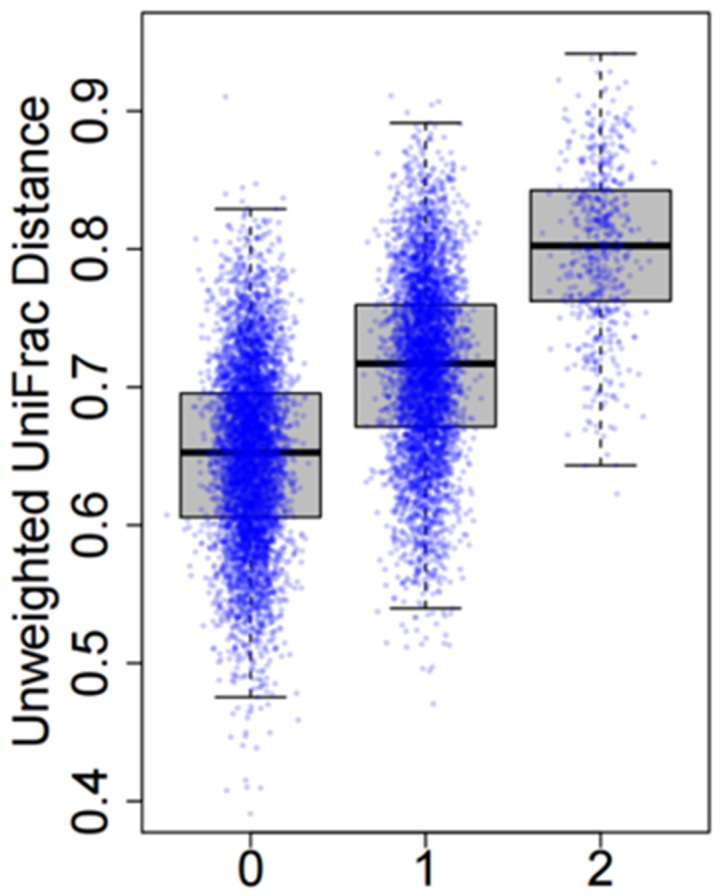
Microbiome distances are positively correlated with genetic distances at an associated SNP.

Intuitively, if the SNP is associated with the microbiome composition, the microbiome distances tend to be smaller for subject pairs with similar genotypic values, as is illustrated in Figure 1. For N subjects, N(N−1)/2 pairs can be divided into three groups with genetic distance 0, 1, and 2. For example, a pair of subjects with genotype (AA, AA) or (BB, BB) has genetic distance 0; a pair of subjects with genotype (AA, BB) or (BB, AA) has genetic distance 2; all other pairs have genetic distance 1. Apparently, we expect the microbiome distance to be positively correlated with genetic distance for subject pairs.

We define $G_{ij}=\left|g_{i}-g_{j}\right|$ as the genetic distance for a pair of subjects (i,j). We assume $d_{ij}=\alpha +\beta_{M}G_{ij}+\varepsilon_{ij}$. A score statistic for testing $H_{0}:\;\beta_{M}=0$ (main effect) vs. $\beta_{M}>0\;$is derived as:

(1) $$S_{M}=\sum_{i<j}d_{ij}^{\prime }G_{ij}\;\;\;\mathrm{with}\;\;d_{ij}^{\prime }=d_{ij}-\frac{2}{N\left(N-1\right)}\sum_{k<l}d_{kl}.\;\;\;\;\;\;\;\;\;\;\;\;\;\;\;\;\;\;$$

The variance $Var_{0}\left(S_{M}|D\right)$ under $H_{0}:\beta_{M}=0$ is calculated by considering the dependence in $\left(G_{ij},G_{kl}\right)$ and conditioning on the distance matrix D. Briefly, we have $Var_{0}\left(S_{M}|D\right)=\sum_{i<j,k<l}d_{ij}^{\prime }d_{kl}^{\prime }Cov\left(G_{ij},G_{kl}\right).$ When (i,j,k,l) are distinct, $G_{ij}$ and $G_{kl}$ are independent; i.e., $Cov\left(G_{ij},G_{kl}\right)=0$. Some algebra leads to

(2) $$\;\;\;\;\;\;\;\;\;\;\;\;\;\;\;\;Var_{0}\left(S_{M}|D\right)=\frac{N\left(N-1\right)}{2}Var\left(G_{ij}\right)\mu_{2}+N\left(N-1\right)\left(N-2\right)Cov\left(G_{ij},G_{ik}\right)\mu_{3}\;\;\;\;\;\;$$

where

(3) $$\mu_{2}=\frac{2}{N\left(N-1\right)}\sum_{i<j}{\left(d_{ij}^{\prime }\right)}^{2}\;$$

and

(4) $$\;\mu_{3}=\frac{2}{N\left(N-1\right)\left(N-2\right)}\sum_{i<j<k}(d_{ij}^{\prime }d_{ik}^{\prime }+d_{ij}^{\prime }d_{jk}^{\prime }+d_{ik}^{\prime }d_{jk}^{\prime }).$$

The details for calculating $Var\left(G_{ij}\right)$ and $Cov\left(G_{ij},G_{ik}\right)$ are in Appendix A. The normalized statistic $Z_{M}=S_{M}/\sqrt{Var_{0}\left(S_{M}|D\right)}\;\sim N\left(0,1\right)$ under $H_{0}$ asymptotically.

In analyses of real data, we typically have to adjust for covariates, including demographic variables and principal component analysis (PCA) scores derived based on genotypes, to eliminate potential population stratification. Given a distance matrix D and v covariates $\left(x_{i1},\cdots ,x_{iv}\right)$, we perform distance-based redundancy analyses using function *capscale* in the *vegan* package [33]. The residual matrix $D^{\prime }$, extracted using the *residuals* function in the *vegan* package [33], is now adjusted for these potential confounding factors and can be used for genetic analysis.

### 2.2. A Score Statistic for Testing Gene–Environment Interaction

Let $E_{i}$ denote an environmental variable. Define $\Delta_{ij}=\left|g_{i}E_{i}-g_{j}E_{j}\right|$. We extend the statistical framework to detect the SNP–environment interaction by assuming $d_{ij}=\alpha +\beta_{M}G_{ij}+\beta_{E}\left|E_{i}-E_{j}\right|+\beta_{I}\Delta_{ij}+\varepsilon_{ij},$ where $\beta_{M}$ denotes the main genetic effect, $\beta_{I}$ denote the additive gene–environment effect, and $\beta_{E}$ denotes the main effect of the environmental factor. We consider testing the null hypothesis that the SNP is not associated with microbiome composition either directly or by interacting with $E,\;i.e.\;H_{0}:\;\beta_{M}=\beta_{I}=0$. The alternative hypothesis is $H_{1}:\;\beta_{M}>0\;\mathrm{or}\;\beta_{I}>0$.

We estimate $\beta_{E}$ and α under $H_{0}$ and calculate $d_{ij}^{\prime }=d_{ij}-\hat{\alpha }-{\hat{\beta }}_{E}\left|E_{i}-E_{j}\right|$. Let $D^{\prime }=\left(d_{ij}^{\prime }\right)$ be the residual matrix. The scores evaluated under $H_{0}$ are $S_{M}=\sum_{i<j}d_{ij}^{\prime }G_{ij}\;\mathrm{for}\;\beta_{M}$ and $S_{I}=\sum_{i<j}d_{ij}^{\prime }\Delta_{ij}$ for $\beta_{I}$. Similar to (2), we derive the variance $Var_{0}\left(S_{I}|D^{\prime }\right)$ by accounting for the dependence in $\left(\Delta_{ij},\Delta_{kl}\right)$:

(5) $$Var_{0}\left(S_{I}|D^{\prime }\right)=\frac{N\left(N-1\right)}{2}Var\left(\Delta_{ij}\right)\mu_{2}+N\left(N-1\right)\left(N-2\right)Cov\left(\Delta_{ij},\Delta_{ik}\right)\mu_{3}.$$

Let $Z_{M}=S_{M}/\sqrt{Var_{0}(S_{M}|D^{\prime })}$ and $Z_{I}=S_{I}/\sqrt{Var_{0}(S_{I}|D^{\prime })}$. Asymptotically, $Z_{M}\sim N\left(0,1\right)$ and $Z_{I}\sim N\left(0,1\right)$ under $H_{0}$. In Appendix B, we derive

(6) $$Cov_{0}\left(S_{M},S_{I}|D^{\prime }\right)=\frac{N\left(N-1\right)}{2}Cov\left(G_{ij},\Delta_{ij}\right)\mu_{2}+N\left(N-1\right)\left(N-2\right)Cov\left(G_{ij},\Delta_{ik}\right)\mu_{3}\;\;\;\;$$

**Figure 2:**
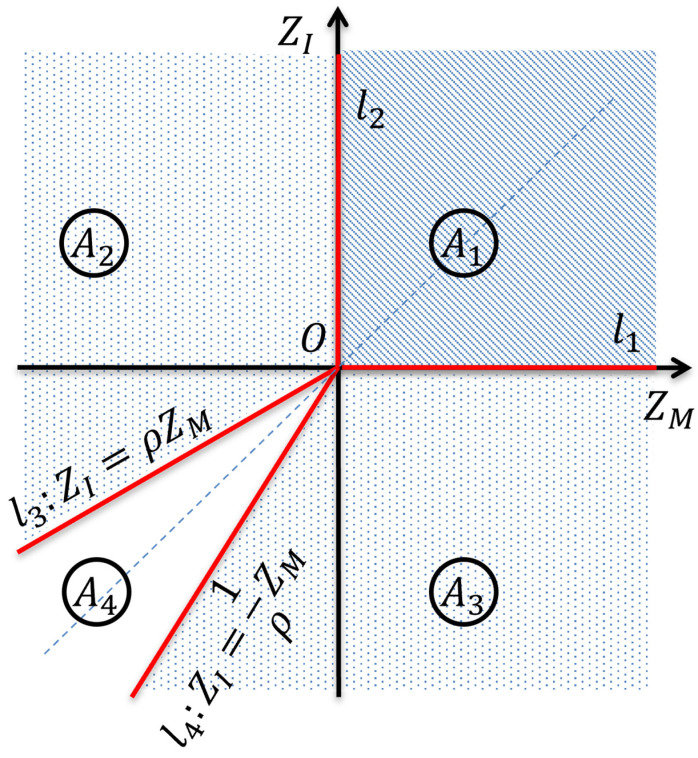
Define the joint test for testing $H_{0}:\;\beta_{M}=\beta_{I}=0$ vs. $\beta_{M}>0\;or\;\beta_{I}>0$. We assume that $Z_{M}\sim N\left(0,1\right)$, $Z_{I}\sim N\left(0,1\right)$ and $cor\left(Z_{M},Z_{I}\right)=\rho$ under $H_{0}$. Details are in Appendix C.

Let $\rho =Cor_{0}\left(Z_{M},Z_{I}|D^{\prime }\right)$ be the correlation between the two statistics. Asymptotically, $\left(Z_{M},Z_{I}\right)$ follows a bivariate normal distribution with a correlation matrix $\Omega =\left(\begin{array}{cc} 1 & \rho \\ \rho & 1 \end{array}\right)$. In Appendix C, we derive a statistic for jointly testing $H_{0}:\;\beta_{M}=\beta_{I}=0$ vs. $H_{1}:\;\beta_{M}>0\;\mathrm{or}\;\beta_{I}>0$. Briefly, the 2D plane is partitioned to four parts (Figure 2). The joint statistic is derived as

(7) $$Q=\{\begin{array}{c} \left(Z_{M},Z_{I}\right)\Omega^{-1}{\left(Z_{M},Z_{I}\right)}^{T}\;\;\;\;\;\;\left(Z_{M},Z_{I}\right)\in A_{1}\;\;\\ {\left(w_{1}Z_{M}+w_{2}Z_{I}\right)}^{2}\;\;\;\;\;\;\;\;\;\;\;\;\;\;\;\left(Z_{M},Z_{I}\right)\in A_{2}\\ {\left(w_{2}Z_{M}+w_{1}Z_{I}\right)}^{2}\;\;\;\;\;\;\;\;\;\;\;\;\;\;\;\left(Z_{M},Z_{I}\right)\in A_{3}\\ 0\;\;\;\;\;\;\;\;\;\;\;\;\;\;\;\;\;\;\;\;\;\;\;\;\;\;\;\;\;\;\;\;\;\;\;\;\;\;\;\;\;\;\;\;\left(Z_{M},Z_{I}\right)\in A_{4} \end{array}$$

where $w_{1}=\left(\theta -1/\theta \;\right)/2$, $w_{2}=\left(\theta +1/\theta \;\right)/2$ and $\theta =\sqrt{\left(1-\rho \right)/\left(1+\rho \right)}$. The asymptotic *p*-value is calculated as

(8) $$P\left(Q>b^{2}\right)=q_{1}P\left(\chi_{2}^{2}>b^{2}\right)+q_{2}P\left(N\left(0,1\right)>b\right)+q_{3}P\left(N\left(0,1\right)>b\right),$$

where $q_{i}=P\left(\left(Z_{M},Z_{I}\right)\in A_{i}\right)$.

### 2.3. Improved p-Value Approximations by Correcting for Skewness and Kurtosis

**Figure 3:**
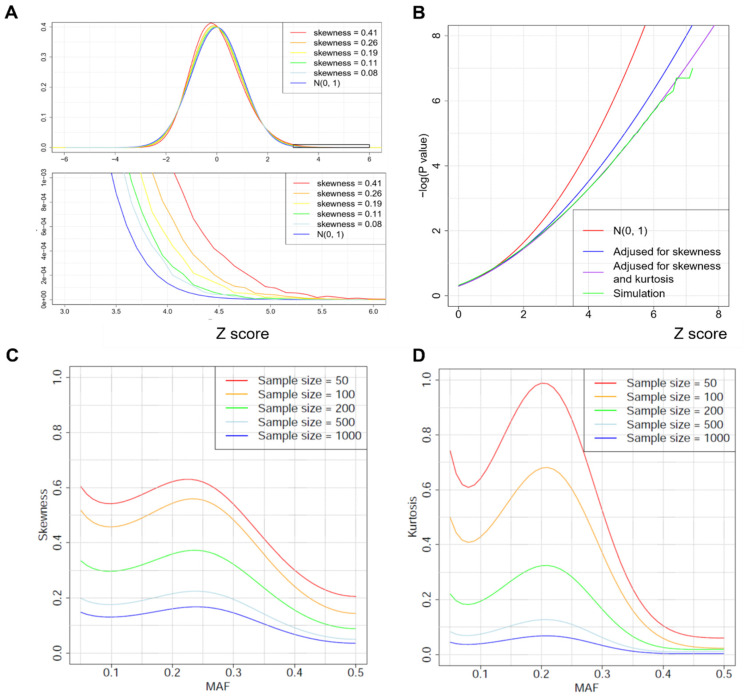
Correcting tail probabilities for skewness and kurtosis. (**A**) The standard normal distribution N(0,1) and an approximately normal distribution with positive skewness. The skewness has big impact when calculating the tail probability P(Z>b) for a large value of b. (**B**) Numerical evaluation of tail probability approximation for $Z_{M}$. We used the unweighted UniFrac distance matrix of 500 samples from the American Gut Project (AGP). For each value of b (>0), we calculated *p*-values $P(Z_{M}>b)$ based on N(0,1), skewness correction, both skewness and kurtosis correction, and 10^8^ simulations. (**C**) Skewness depends on minor allele frequency (MAF) of SNPs and the sample size of the study, calculated based on the weighted UniFrac distance matrix in AGP data. (**D**) Kurtosis depends on MAF of SNPs and the sample size, calculated based on the weighted UniFrac distance matrix in the AGP data.

Theoretic investigation suggests that the score statistics $Z_{M}$ and $Z_{I}\;$ have a positive skewness, which makes the tail probability approximations based on the asymptotic distribution N(0,1) unacceptably liberal (Figure 3A,B). In a numeric example with skewness γ=0.2, P(Z>5)=2.9 × 10^−7^ based on N(0, 1), which is approximately two orders of magnitude more significant than *p* = 3.9 × 10^−5^ based on 10^8^ permutations. The significance inflation becomes worse for smaller *p*-values and larger skewness γ. Similar but more tedious calculations suggest that both statistics have positive kurtosis, making the approximation based on N(0, 1) even worse. One possible solution is to approximate tail probabilities using permutations or bootstrap. However, these resampling methods are computationally prohibitive for testing millions of common SNPs in a large-scale study.

To address this problem, we calculated the skewness γ and kurtosis κ of the score statistics under $H_{0}\;$(Appendix D). We propose to improve the tail probability approximation $P_{0}\left(Z>b\right)\;$ by correcting for the skewness and kurtosis, following the skewness correction in linkage analysis [34,35]. Technical details are provided in Appendix E. Correcting for both skewness and kurtosis leads to an approximation

(9) $$P_{0}\left(Z>b\right)\approx e^{-b\xi_{1}+\left(1+\sigma_{1}^{2}\right)\xi_{1}^{2}/2+\gamma \xi_{1}^{3}/6+\kappa \xi_{1}^{4}/24\;}\Phi \left(-\sigma_{1}\xi_{1}\right)$$

where $\xi_{1}$ satisfies $\xi +\gamma \xi^{2}/2+\kappa \xi^{3}/6=b$, $\sigma_{1}^{2}=1+\gamma \xi_{1}+\kappa \xi_{1}^{2}/2$ and Φ(·) is the cumulative distribution function of N(0,1). Correcting for skewness but ignoring kurtosis (i.e., assuming κ=0) leads to an approximation

(10) $$P_{0}\left(Z>b\right)\approx e^{-b\xi_{2}+\left(1+\sigma_{2}^{2}\right)\xi_{2}^{2}/2+\gamma \xi_{2}^{3}/6\;}\Phi \left(-\sigma_{2}\xi_{2}\right)$$

where $\xi_{2}=\left(\sqrt{1+2\gamma b}-1\right)/\gamma$, $\sigma_{2}^{2}=1+\gamma \xi_{2}$. Numerical results presented in Figure 3B demonstrate that (9) works very well.

Given the distance matrix D, $\gamma_{M}\propto 1/N^{1/2}$, $\gamma_{I}\propto 1/N^{1/2\;}$, $\kappa_{M}\propto 1/N$ and $\kappa_{I}\propto 1/N$ (Appendix D). Thus, skewness decays much more slowly with sample size N than kurtosis (Figure 3C,D). Thus, even for a large study with thousands of samples, correcting for skewness is necessary for accurately evaluating the tail probabilities. Importantly, both skewness and kurtosis highly depend on the MAF, suggesting that the impact of skewness and kurtosis is different across SNPs with a different MAF. Numerical studies (Figure 3C,D) show that skewness and kurtosis are minimized when MAF = 0.5 and maximized when MAF ≈ 0.2–0.3.

Finally, we discuss how to approximate the tail probability of Q in (7) for testing $H_{0}:\;\beta_{M}=\beta_{I}=0\;$by correcting for non-normality in $Z_{M}$ and $Z_{I}$. When $\left(Z_{M},Z_{I}\right)\in A_{2}$ (or $A_{3}$), we calculate the skewness $E{\left(w_{1}Z_{M}+w_{2}Z_{I}\right)}^{3}$ and the kurtosis $E{\left(w_{1}Z_{M}+w_{2}Z_{I}\right)}^{4}-3$ and use (9) to approximate $P(w_{1}Z_{M}+w_{2}Z_{I}>b)$. When $\left(Z_{M},Z_{I}\right)\in A_{1}$, we first approximate their marginal *p*-values as $p_{M}$ and $p_{I}$ by (9), and then calculate the normal quantile $z_{M}=\Phi \left(1-p_{M}\right)$ and $z_{I}=\Phi \left(1-p_{I}\right)$. Because the correction primarily impacts the tails of the distributions, the correlation between the two statistics will remain roughly unchanged; i.e., $cor_{0}\left(Z_{M},Z_{I}\right)\approx cor_{0}(z_{M},z_{I})$. Thus, when $\left(Z_{M},Z_{I}\right)\in A_{1}$, the tail probability is approximated as $P(\chi_{2}^{2}>\left(z_{M},z_{I}\right)\Omega^{-1}\left(z_{M},z_{I}\right)\prime )$.

## 3. Results

### 3.1. Simulation Results

The main purpose of simulations was to investigate the type-I error of $Z_{M}$ (for testing the main genetic effect), $Z_{I}$ (for detecting SNP–environment interactions), and Q (for detecting either the main genetic effect or SNP–environment effect or both). Simulations were performed under different combinations of sample size, MAF, and microbiome distance matrices. To make the simulations realistic, we used an unweighted distance matrix of the fecal microbiome samples with the 16S rRNA V4 region sequences from the American Gut Project (AGP) [36]. The OTU table, rarefied to 10,000 sequence reads per sample, as well as the metadata were downloaded from the AGP website. Samples with less than 10,000 sequence reads were excluded from the analysis. The weighted and the unweighted UniFrac distance matrices were generated in the Quantitative Insights Into Microbial Ecology [21] (QIIME) pipeline. Because antibiotics may substantially change the microbiome composition to generate outliers that may distort the null distribution, we excluded samples with self-reported history of antibiotic usage within one month. After quality control, 1879 subjects remained for analysis. In the simulations, we randomly selected N samples for a given sample size N.

For each setting, the type-I error rates were evaluated based on 10^8^ simulations under $H_{0}$. For the interaction test and the joint test, the binary environment factor had a frequency of 50% and was simulated independent of the SNP. The type-I error rates are summarized in Table 1 for the weighted UniFrac distance matrix. The skewness and kurtosis are reported in Figure 3C,D. The statistics adjusted for skewness and kurtosis have accurate type-I error rates while the statistics without adjustment have unacceptably high type-I error rates. As the sample size increases, the impact of skewness and kurtosis decreases. However, even for a study with N=1000, the type-I error rates are still seriously inflated. The results for the unweighted UniFrac distance matrix and for MAF = 0.5 are reported in Table S1.

### 3.2. Software Implementation, Memory Requirement, and Computational Complexity

**Figure 4:**
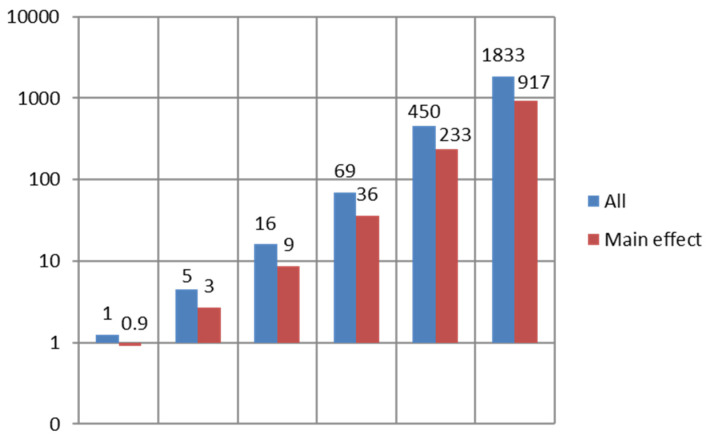
Computation time for a microbiome GWAS with 500,000 SNPs. “Main”: computation time for testing main effect only. “All”: computation time for testing main effect, interaction and the joint null hypothesis $H_{0}:\;\beta_{M}=0,\beta_{I}=0$.

We implemented our algorithms in a software package, microbiomeGWAS, which is freely available at https://github.com/lsncibb/microbiomeGWAS [30], accessed on 30 May 2022. MicrobiomeGWAS requires three sets of files: a microbiome distance matrix file, a set of PLINK binary files for GWAS genotypes, and a set of covariates. MicrobiomeGWAS processes one SNP at a time and does not load all genotype data into memory; thus, it requires only memory for storing the distance matrix. Variance, skewness, and kurtosis can be partitioned into two parts related with the microbiome distance matrix and the MAF of the SNP separately; thus, we can quickly calculate these quantities for a predefined grid of MAFs. The overall computational complexity is about $O(N^{2}M)$, where N is the sample size and M is the number of SNPs. Figure 4 reports the computation time on a Linux server using a single core. For a study with 10,000 subjects, it takes approximately 15 h for analyzing the main effect and approximately 30 h for analyzing both the main and interaction effects for 0.5 million variants. As a comparison, in a recent microbiome GWAS [37], to analyze 7 × 10^−6^ variants for the main effect and *n* = 3382 subjects in the SHIP-TREND cohort [37], it would take 61 years using one CPU and 94 days using one graph-processing unit for parallel computation. Moreover, their analytic pipeline could not jointly analyze all 8956 subjects from five cohorts because of the computational burden; instead, they performed a stepwise search that may cause power loss.

### 3.3. GWAS of Microbiome Diversity in Adjacent Normal Lung Tissues

We applied our methods to a set of lung cancer patients of Italian ancestry in the EAGLE [31] study. All subjects have germline genome-wide SNPs [32] and 16S rRNA microbiome data (V3-V4 region, Illumina MiSeq, 300 paired-end) in histologically normal lung tissues from these patients. Here, the histologically normal lung tissues were 1~5 cm from the tumor tissue. We performed a series of quality control steps to filter out low-quality sequence reads: average quality score <20 over 30 bp windows, less than 60% similarity to the Greengenes [38] reference, or identified as chimera reads using UCHIME [39]. Sequence reads were then processed by QIIME [21] to produce the relative abundances (RA) of taxa, two α-diversity metrics (observed number of species and Shannon’s index), and β diversity metrics (unweighted and weighted UniFrac distances) rarified to 1000 reads. We included 147 subjects with at least 1000 high-quality sequence reads for genetic association analysis.

Out of the 147 subjects, 78 are current smokers, 8 never smoked, and 61 are former smokers. Because of the small number of never smokers, we merged never and former smokers as non-current smokers. All of the genetic association analyses were adjusted for sex, age, smoking status, and the top three PCA scores derived based on genome-wide SNPs. Here, the top three PCA scores were selected for controlling population stratification because the other PCA scores were unassociated with the distance matrices. We included 383,263 common SNPs with MAF ≥ 10% because rarer SNPs were expected to have no statistical power given the current sample size. We first performed GWAS analysis using PLINK [40] to identify the SNPs associated with taxa with an average RA greater than 0.1% or two α-diversity metrics. We did not detect genome-wide significant associations with either the main effects or gene–smoking interactions.

**Figure 5:**
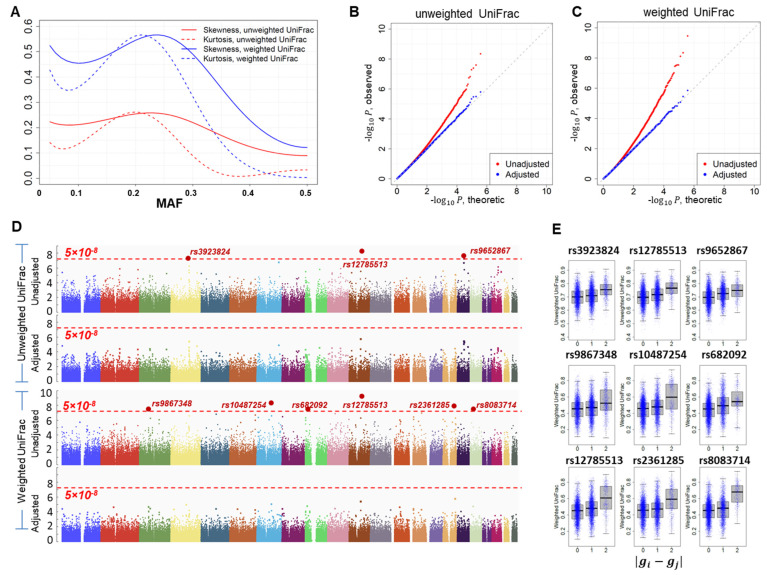
Results of analyzing the microbiome GWAS data of 147 adjacent normal lung tissues in the EAGLE study. (**A**) Skewness and kurtosis for the main effect test using the unweighted and the weighted UniFrac distance matrices. (**B**) Quantile–quantile (QQ) plot for association *p*-values using the unweighted UniFrac distance matrix. “Adjusted”: *p*-values were corrected for skewness and kurtosis. “Unadjusted”: *p*-values were approximated based on the asymptotic distribution N(0,1). (**C**) Quantile–quantile (QQ) plot for association *p*-values using the weighted UniFrac distance matrix. (**D**) Manhattan plots based on the unweighted or the weighted UniFrac distance matrices. (**E**) Box plots for the top nine loci in microbiome GWAS analysis. Subject pairs are classified into three groups according to the genetic distance $\left|g_{i}-g_{j}\right|$ at the SNP. The y-coordinate is the microbiome distance.

Next, we performed GWAS analysis using unweighted and weighted UniFrac distance matrices as a representation of eubacteria β diversity. The results for testing the main effects are reported in Figure 5. Results for testing the joint effects (main effect and SNP by smoking status interaction) are reported in Figure S1. Because of the small sample size, we observed large values of skewness and kurtosis, with the magnitude varying with the MAF of the SNPs (Figure 5A). The score statistics based on the weighted UniFrac distance matrix had a much larger skewness and kurtosis than did the unweighted UniFrac matrix. Figure 5B,C report the quantile–quantile (QQ) plot of the logarithm of the association *p*-values for the unweighted and weighted UniFrac distance matrices, respectively. For each distance matrix, we produced QQ plots for *p*-values based on the asymptotic approximation and for *p*-values adjusted for skewness and kurtosis. For both distance matrices, the QQ plots before adjustment strongly deviated from the expected uniform distribution. Our adjustment eliminated the deviation. In addition, consistent with the observation that the skewness and kurtosis were larger for the weighted UniFrac distance matrix, the QQ plot deviated more for the analysis based on the weighted UniFrac distance. Note that the skewness and kurtosis only affect the tail probabilities; thus, the inflation of the QQ plot is not reflected by the genomic control lambda value [41], calculated as the median of the *p*-values. In fact, lambda ≈1 for all four QQ plots.

Without correcting for skewness and kurtosis, we identified three and six loci achieving genome-wide significance ($p<5\times 10^{-8}$) for the unweighted and weighted UniFrac distance matrices, respectively (Figure 5D). After correcting for skewness and kurtosis, no locus remained genome-wide significant (Figure 5D), which was verified by 10^8^ permutations. Importantly, skewness and kurtosis had a dramatic effect on tail probabilities. Here, we use SNP rs12785513 as an example, which was identified as the top SNP in both analyses. In the unweighted UniFrac analysis, *p* = 4.4 × 10^−9^ without adjustment and *p* = 1.6 × 10^−6^ after adjustment, a 364-fold inflation. The inflation was even larger for weighted UniFrac analysis because of larger skewness and kurtosis (Figure 5A). In fact, *p* = 3.4 × 10^−10^ without adjustment and *p* = 3.5 × 10^−6^ after adjustment, a 1000-fold inflation. Although these SNPs were not significant genome-wide, they were the top SNPs from the current study. Thus, we report box-plots for each of these nine SNPs (Figure 5E). As expected, in all box plots, microbiome distances tend to be larger in subject pairs with greater genetic distance at these SNPs. These associations remain to be replicated in studies with larger sample sizes.

Finally, we concentrated on the six common SNPs in four genomic regions reported to be associated with lung cancer risk in GWAS of European subjects: rs2036534 and rs1051730 at 15q25.1 [42,43,44,45] (*CHRNA5*–*CHRNA3*–*CHRNB4*), rs2736100 and rs401681 at locus 5p15.33 [31,46] (*TERT*/*CLPTM1L*), rs6489769 [47] at 12p13.3 (*RAD52*), and rs1333040 at 9p21.3 [48] (*CDKN2A*/*CDKN2B*). The SNPs at 15q25.1 and 5p15.33 have the largest effect sizes for lung cancer risk based on the meta-analysis from the Transdisciplinary Research in Cancer of the Lung (TRICL) consortium [48]: OR = 1.32 for rs1051730, OR = 1.26 for rs2036534, OR = 1.13 for rs2736100, and OR = 1.14 for rs401681. Rs3131379 at locus 6p21.33 [46] (*BAT3*/*MSH5*) was excluded because the MAF = 7.5%. No SNPs were significantly associated with taxa RAs or α-diversity metrics after correcting for multiple testing. However, association analysis based on the UniFrac distance matrices provided evidence that these SNPs may be associated with the lung microbiota (Table 2). These SNPs were independent except that rs2036534 and rs1051730 at 15q25.1 were weakly correlated with R^2^ = 0.15. A test combining six z-scores ($Z_{M}$) and adjusting for the weak correlation yielded overall *p*-values of 0.0033 and 0.011 for the unweighted and the weighted UniFrac distance matrices, respectively. These results suggest that lung cancer risk SNPs were enriched for genetic association with the composition of the lung microbiome. The results for testing interactions and joint effects are reported in Table S2.

## 4. Discussion

We developed a software package, microbiomeGWAS, for identifying host genetic variants associated with microbiome composition. MicrobiomeGWAS can test both the main effect and SNP–environment interactions. Importantly, we found that the score statistics had positive skewness and kurtosis and that the tail probabilities evaluated based on asymptotic approximations were very liberal. We addressed this problem by explicitly adjusting for skewness and kurtosis. MicrobiomeGWAS runs very efficiently and takes only 36 min for testing main effects and 69 min for testing joint effects in a GWAS with 2000 subjects and 500,000 markers. Other statistical methods exist for testing the association of microbiome distance matrices. PERMANOVA [27] is an extension of multivariate analysis of variance to a matrix of pairwise distances and relies on permutations to evaluate significance. MiRKAT [49], a recently proposed method based on kernel regression, takes hours for evaluating one association for 2000 subjects. Neither is computationally feasible for analyzing a large-scale GWAS of a microbiome. Recently, an asymptotic distribution was proposed to approximate the *p*-value for the PERMANOVA pseudo-F statistic [50]; however, whether it is sufficiently accurate for very small *p*-values (*p* < 5 $\times \;10^{-8},\;\mathrm{for}\;\mathrm{GWAS}$) remains to be investigated.

Interactions of host genetic susceptibility with the microbiome have been postulated for many conditions, including inflammatory bowel diseases [51,52], autoimmune and rheumatic diseases [53,54,55,56], diabetes [57], and cancer, especially of the colon [58]. All models of these host–microbiome interactions also note the critical role of environmental factors, including diet, smoking, drugs, and antibiotics and other medications [59]. Although based on a very small initial sample set, the suggestive associations that we found between the six known lung cancer risk SNPs and the microbiome of adjacent normal lung tissue samples, including effects of cigarette smoking, provide preliminary evidence that our microbiomeGWAS method is likely to be a useful tool for generating data that will unravel host–microbiome interactions with high confidence.

We are working on two extensions for microbiomeGWAS: (1) jointly testing additive and dominant effects; and (2) testing genetic associations using many microbiome distance matrices. We have assumed an additive effect model (Figure 1); however, several top SNPs in the EAGLE data suggest a dominant effect (e.g., rs8083714 in Figure 5E). Thus, a statistic for jointly testing the additive and dominant effects might be powerful for this scenario. The second extension is motivated by the fact that that the power to detect associations depends heavily on the choice of distance matrix. The recently developed generalized UniFrac [26] (gUniFrac) defines a series of distance matrices to reflect the different emphases of using taxa relative abundance information. gUniFrac has been shown to have a robust power for association studies [26]. Extending microbiomeGWAS to gUniFrac, however, requires solving two problems. First, the computational complexity is proportional to the number of distance matrices analyzed for associations, which can be addressed by implementing the algorithms using multithreading technology. Second, we need to derive accurate analytic approximations to the association *p*-values by correcting for the multiple testing introduced by many distance matrices. MiRKAT [49] has an option for using gUniFrac; however, intensive permutations are required to evaluate *p*-values.

In summary, GWAS of the microbiome of each body site has the potential to help one understand microbiome variation, to elucidate the biological mechanisms of genetic associations, to improve the power of identifying novel disease-associated genetic variants, and to improve the performance of genetic risk prediction. We expect our methods and software to be useful for large-scale GWAS of the human microbiome.

## Figures and Tables

**Table 1:** Type-I error rates estimated based on 10^8^ simulations. Minor allele frequency = 20%. Simulations were based on the weighted UniFrac distance matrix of the gut microbiome data from the American Gut Project. Reported are the type-I error inflation factor. A value greater than 1 indicates an inflated type-I error.

		*Z_M_*			*Z_I_*			*Q*		
	N	α = 10^−3^	10^−5^	10^−7^	10^−3^	10^−5^	10^−7^	10^−3^	10^−5^	10^−7^
Asymptotic approximation	100	5.5	51.6	610.0	4.7	36.1	342.8	7.3	80.9	1148.0
	200	3.7	23.0	187.3	3.1	15.8	105.5	4.6	33.0	316.7
	500	2.4	9.4	45.2	2.1	6.7	25.5	2.8	11.9	64.1
	1000	2.0	5.7	21.3	1.8	4.4	14.0	2.2	6.9	28.5
Adjusted for skewness and kurtosis	100	1.0	1.2	0.7	1.0	1.1	0.6	1.0	1.5	2.0
	200	1.0	1.1	1.0	1.0	1.1	0.7	0.9	1.3	1.8
	500	1.0	1.1	1.3	1.0	1.0	0.9	0.9	1.0	1.7
	1000	1.0	1.0	1.2	1.0	1.0	0.8	0.9	1.0	1.1

**Table 2:** Association *p*-values between lung cancer risk SNPs and microbiome composition in the EAGLE data.

SNP	Chr	Annotated Genes	Unweighted UniFrac	Weighted UniFrac
rs2036534	15q25.1	*CHRNA3/4/5*	0.425	0.167
rs1051730	15q25.1	*CHRNA3/4/5*	0.020	0.401
rs2736100	5p15.33	*TERT*	0.089	0.267
rs401681	5p15.33	*CLPTM1L*	0.056	0.005
rs6489769	12p13.3	*RAD52*	0.197	0.329
rs1333040	9p21.3	*CDKN2A*/*B*	0.249	0.224
Overall test			0.0032	0.011

## Data Availability

The genetic data for the EAGLE study can be accessed from dbGap with accession number phs000093.v2.p2. The American Gut Project data used for simulations can be obtained from https://github.com/biocore/American-Gut (accessed on 25 May 2022).

## Supplementary Materials

The following supporting information can be downloaded at: https://www.mdpi.com/article/10.3390/genes13071224/s1, Table S1: Type-I error rates estimated based on 10^8^ simulations. Table S2: Association P-values between lung cancer risk SNPs and microbiome composition in the EAGLE data. Figure S1: Quantile-quantile (QQ) plot for association *p*-values testing the joint effects (main effect and SNP by smoking interaction) using the unweighted UniFrac distance matrices. Figure S2: Derivation of the likelihood ratio statistic Q in (7) and (8). Figure S3: Calculations related with genetic dependence.

## Appendix A. Calculating Calculating $Var\left(G_{ij}\right)$, $Cov\left(G_{ij},\;G_{ik}\right)$, $Var\left(\Delta_{ij}\right)$ and $Cov\left(\Delta_{ij},{\;\Delta }_{ik}\right)$

We first calculate $E\left(G_{ij}\right)$, $Var\left(G_{ij}\right)$, and $Cov\left(G_{ij},\;G_{ik}\right)$. Let $p_{t}=P\left(g_{i}=t\right)$ with $p_{0},p_{1},p_{2}\geq 0\;$and $p_{0}+p_{1}+p_{2}=1$. We can also assume the Hardy–Weinberg equilibrium and characterize the probabilities as the allele frequency: $p_{0}={\left(1-f\right)}^{2},p_{1}=2f\left(1-f\right)$ and $p_{2}=f^{2}$. Some algebra leads to

(A1) $$E\left(G_{ij}\right)=E\left|g_{i}-g_{j}\right|=\sum_{m,n\in \left\{0,\;1,\;2\right\}}p_{m}p_{n}\left|m-n\right|=2p_{0}p_{1}+2p_{1}p_{2}+4p_{0}p_{2}\;\;\;\;\;\;\;$$

(A2) $$Var\left(G_{ij}\right)=E\left(G_{ij}^{2}\right)-E{\left(G_{ij}\right)}^{2}=\left(2p_{0}p_{1}+2p_{1}p_{2}+8p_{0}p_{2}\right)-{\left(2p_{0}p_{1}+2p_{1}p_{2}+4p_{0}p_{2}\right)}^{2}\;\;$$

(A3) $$\;\;\;\;Cov\left(G_{ij},\;G_{ik}\right)=p_{1}\left(1-p_{1}\right)+4p_{0}p_{2}\left(1+p_{1}\right)-{\left(2p_{0}p_{1}+2p_{1}p_{2}+4p_{0}p_{2}\right)}^{2}\;\;\;\;\;$$

Now consider $\Delta_{ij}=\left|g_{i}E_{i}-g_{j}E_{j}\right|$. When $E_{i}$ is binary, $g_{i}E_{i}=0,\;1$ or 2. Let $p_{t}^{\prime }=P\left(g_{i}E_{i}=t\right)$. Then, $E\left(\Delta_{ij}\right)$, $Var\left(\Delta_{ij}\right)$, and $Cov\left(\Delta_{ij},{\;\Delta }_{ik}\right)$ can be calculated similarly using (A1)–(A3).

## Appendix B. Calculating $\rho =Cor_{0}(Z_{M},Z_{I}|D\prime )$

Let $G_{ij}^{\prime }=G_{ij}-EG_{ij}$ and $\Delta_{ij}^{\prime }=\Delta_{ij}-E\Delta_{ij}$. We first calculate covariance under $H_{0}$:

$$Cov_{0}\left(S_{M},S_{I}|D^{\prime }\right)=Cov_{0}\left(\sum_{i<j}d_{ij}^{\prime }G_{ij}^{\prime },\sum_{m<n}d_{mn}^{\prime }\Delta_{mn}^{\prime }\right)=\sum_{i<j,m<n}d_{ij}^{\prime }d_{mn}^{\prime }Cov\left(G_{ij},\Delta_{mn}\right).$$

When (i,j,m,n) are distinct, $Cov\left(G_{ij},\Delta_{mn}\right)=0$. Some algebra leads to

(A4) $$\;\;\;\;\;\;\;\;\;\;\;\;\;\;\;\;\;\;\;\;\;\;\;\;\;\;\;\;\;\;\;\;\;\;Cov_{0}\left(S_{M},S_{I}|D^{\prime }\right)=\left(\begin{array}{c} N\\ 2 \end{array}\right)Cov\left(G_{ij},\Delta_{ij}\right)\mu_{2}+6\left(\begin{array}{c} N\\ 3 \end{array}\right)Cov\left(G_{ij},\Delta_{ik}\right)\mu_{3}$$

with $\mu_{2}$ and $\mu_{3}$ specified in (3) and (4). Combining (2), (5) and (A4), we have

(A5) $$\;\;\;\;\;\;\;\;\;\;\;\;\;\;\;\;\;\;\;\rho =\frac{Cov_{0}\left(S_{M},S_{I}|D^{\prime }\right)}{\sqrt{Var_{0}\left(S_{M}|D^{\prime }\right)Var_{0}\left(S_{I}|D^{\prime }\right)}}\overset{N\to \infty }{\to }\frac{Cov\left(G_{ij},\Delta_{ik}\right)}{\sqrt{Cov\left(G_{ij},G_{ik}\right)Cov\left(\Delta_{ij},\Delta_{ik}\right)}}\;\;\;\;\;\;\;\;\;\;\;\;\;\;\;\;\;\;\;\;\;\;\;\;\;$$

Equation (A5) suggests that the correlation is asymptotically independent of the microbiome distance matrix. In the real-data analyses, we found that (A5) was very accurate when sample size N≥50. The details of calculating $Cov\left(G_{ij},\Delta_{ij}\right)$ and $Cov\left(G_{ij},\Delta_{ik}\right)$ are provided in Supplemental Data.

## Appendix C. A Statistic for Testing $H_{0}:\beta_{M}=\beta_{I}=0\;\mathrm{vs}.\;H_{1}:\beta_{M}>0\;\mathrm{or}\;\beta_{I}>0$

Denote $Z={\left(Z_{M},\;Z_{I}\right)}^{T}$. Under $H_{0}$, Z~N(0, Σ) with $\Sigma =\left(\begin{array}{cc} 1 & \rho \\ \rho & 1 \end{array}\right)$. Let $\xi_{M}=E_{1}Z_{M}\geq 0$ and $\xi_{I}=E_{1}Z_{I}\geq 0$ be the non-centrality parameter of the two score statistics. Apparently, the original testing problem is equivalent for testing $H_{0}:\xi_{M}=\xi_{I}=0\;\mathrm{vs}.\;H_{1}:\xi_{M}>0\;\mathrm{or}\;\xi_{I}>0$. Given the observed values $\left(Z_{M},Z_{I}\right)$, the likelihood ratio statistic is simplified as

(A6) $$Q=Z^{T}\Sigma^{-1}Z-{\left(Z-\xi \right)}^{T}\Sigma^{-1}\left(Z-\xi \right)$$

where $\xi ={\left(\xi_{M},\xi_{I}\right)}^{T}={\mathrm{arginf}}_{\xi_{M}\geq 0,\xi_{I}\geq 0}Q$ (Figure S2A).

To simplify the optimization problem in (A6), we perform a linear transformation: $Y^{T}=Z^{T}\Sigma^{-\frac{1}{2}}\;$and $v^{T}=\xi^{T}\Sigma^{-\frac{1}{2}}$, where

(A7) $$\Sigma^{-\frac{1}{2}}=\frac{1}{\sqrt{2}}\left(\begin{array}{cc} 1 & 1\\ -1 & 1 \end{array}\right)\left(\begin{array}{cc} 1/\sqrt{1-\rho } & 0\\ 0 & 1/\sqrt{1+\rho } \end{array}\right)$$

Under this transformation, $Q=Y^{T}Y-{\left(Y-v\right)}^{T}\left(Y-v\right)$ and can be interpreted as the difference of the square of two distances (Figure S2B). The original parameter space $\left\{\left(\xi_{M},\xi_{I}\right):\xi_{M}\geq 0,\xi_{I}\geq 0\right\}$ is now transformed to $\left\{\left(\nu_{1},\nu_{2}\right):\nu_{2}\geq \theta \nu_{1},\nu_{2}\geq -\theta \nu_{1}\right\}$ with $\theta =\sqrt{\left(1-\rho \right)/\left(1+\rho \right)}$. Thus, the new parameter space is bounded by two lines represented by $\nu_{2}\geq \theta \nu_{1}$ and $\nu_{2}\geq -\theta \nu_{1}$. We partition the 2D plane into four parts (see Figure S2B), identify $v={\mathrm{arginf}}_{v\in A_{1}}{\left(Y-v\right)}^{T}\left(Y-v\right)$ and calculate Q:

(A8) $$Q=\{\begin{array}{c} {Y_{1}}^{2}+{Y_{2}}^{2}\;\;\;\;\;\;\;\;\;\;\;\;\;\;\;\;\;\;\;\;\;\;\;\;\;\;\;\;\;\;\;\;\;\;\;\;\;\;\;\;\left(Y_{1},Y_{2}\right)|\in A_{1}\\ {\left(Y_{2}-Y_{1}/\theta \right)}^{2}/\left(1+\theta^{-2}\right)\;\;\;\;\;\;\;\;\;\;\;\;\;\;\;\left(Y_{1},Y_{2}\right)|\in A_{2}\;\;\\ {\left(Y_{2}+Y_{1}/\theta \right)}^{2}/\left(1+\theta^{-2}\right)\;\;\;\;\;\;\;\;\;\;\;\;\;\;\;\left(Y_{1},Y_{2}\right)|\in A_{3}\;\;\\ 0\;\;\;\;\;\;\;\;\;\;\;\;\;\;\;\;\;\;\;\;\;\;\;\;\;\;\;\;\;\;\;\;\;\;\;\;\;\;\;\;\;\;\;\;\;\;\;\;\;\;\;\;\;\left(Y_{1},Y_{2}\right)|\in A_{4}\;\; \end{array}$$

We now perform an inverse transformation using matrix

(A9) $$\;\;\;\;\;\Sigma^{\frac{1}{2}}=\left[\frac{1}{\sqrt{2}}\left(\begin{array}{cc} \sqrt{1-\rho } & 0\\ 0 & \sqrt{1+\rho } \end{array}\right)\left(\begin{array}{cc} 1 & -1\\ 1 & 1 \end{array}\right)\right]\;\;$$

to return to the original parameter space. The four areas $\left\{A_{1},A_{2},A_{3},A_{4}\right\}$ under the original space are in Figure 2 and Figure S2C.

Tedious calculations show that ${\left(Y_{2}+Y_{1}/\theta \right)}^{2}/\left(1+\theta^{-2}\right)={\left(w_{2}Z_{M}+w_{1}Z_{I}\right)}^{2}\;$with $w_{1}=\left(\theta -1/\theta \right)/2$ and $w_{2}=\left(\theta +1/\theta \right)/2$. Similarly, ${\left(Y_{2}-Y_{1}/\theta \right)}^{2}/\left(1+\theta^{-2}\right)={\left(w_{1}Z_{M}+w_{2}Z_{I}\right)}^{2}$. This proves (7). In addition, $w_{1}Z_{M}+w_{2}Z_{I}\geq 0$ and $w_{1}^{2}+2\rho w_{1}w_{2}+w_{2}^{2}=1$; thus, $P\left\{{\left(w_{1}Z_{M}+w_{2}Z_{I}\right)}^{2}>b^{2}\right\}=P\left\{w_{1}Z_{M}+w_{2}Z_{I}>b\right\}=P\{N\left(0,1\right)>b\}$. This proves (8). The probabilities in (8) could also be calculated from Figure S2B: $q_{1}=1/2-\left(\arctan\theta \right)/\pi \;,q_{2}=q_{3}=1/4$.

## Appendix D. Calculating Skewness and Kurtosis under $H_{0}$

By definition, $\gamma =E_{0}\left(S_{M}^{3}|D^{\prime }\right)/Var_{0}^{3/2\;}\left(S_{M}|D^{\prime }\right)$ and $\kappa =E_{0}\left(S_{M}^{4}|D^{\prime }\right)/Var_{0}^{2\;}\left(S_{M}|D^{\prime }\right)-3$. We first calculate $E_{0}\left(S_{M}^{3}|D^{\prime }\right)$. Let $G_{ij}^{\prime }=G_{ij}-EG_{ij}$. We have

$$E_{0}\left(S_{M}^{3}|D^{\prime }\right)=E_{0}{\left(\sum_{i<j}d_{ij}^{\prime }G_{ij}^{\prime }\right)}^{3}=\sum_{i<j,\;m<n,s<t}d_{ij}^{\prime }d_{mn}^{\prime }d_{st}^{\prime }EG_{ij}^{\prime }G_{mn}^{\prime }G_{st}^{\prime }.$$

Figure S3A lists all combinations of (i,j,m,n,s,t) with $EG_{ij}^{\prime }G_{mn}^{\prime }G_{st}^{\prime }\neq 0$; then

$$E_{0}\left(S_{M}^{3}|D^{\prime }\right)=\left(\begin{array}{c} N\\ 2 \end{array}\right)\mu_{4}EG_{ij}^{\prime 4}+\left(\begin{array}{c} N\\ 3 \end{array}\right)\left(\mu_{5}EG_{ij}^{\prime 2}G_{ik}^{\prime }+\mu_{6}EG_{ij}^{\prime }G_{jk}^{\prime }G_{ik}^{\prime }\right)+\left(\begin{array}{c} N\\ 4 \end{array}\right)\left(\mu_{7}EG_{ij}^{\prime }G_{jk}^{\prime }G_{kl}^{\prime }+\mu_{8}EG_{ij}^{\prime }G_{ik}^{\prime }G_{il}^{\prime }\right),$$

where $\left(\mu_{4},\mu_{5},\mu_{6},\mu_{7},\mu_{8}\right)$ are provided in Supplemental Data. Similarly,

$$E_{0}\left(S_{M}^{4}|D^{\prime }\right)=E_{0}{\left(\sum_{i<j}d_{ij}^{\prime }G_{ij}^{\prime }\right)}^{4}=\sum_{i<j,\;m<n,s<t,x<y}d_{ij}^{\prime }d_{mn}^{\prime }d_{st}^{\prime }d_{xy}^{\prime }EG_{ij}^{\prime }G_{mn}^{\prime }G_{st}^{\prime }G_{xy}^{\prime }.$$

Figure S3B lists combinations of (i,j,m,n,s,t,x,y) with $EG_{ij}^{\prime }G_{mn}^{\prime }G_{st}^{\prime }G_{xy}^{\prime }\neq 0$. Thus,

$$\begin{array}{c} E_{0}\left(S_{M}^{4}|D\right)=\left(\begin{array}{c} N\\ 2 \end{array}\right)\mu_{9}EG_{ij}^{\prime 4}+\left(\begin{array}{c} N\\ 3 \end{array}\right)\left(\mu_{10}EG_{ij}^{\prime 3}G_{ik}^{\prime }+\mu_{11}EG_{ij}^{\prime 2}G_{ik}^{\prime 2}+\mu_{12}EG_{ij}^{\prime 2}G_{jk}^{\prime }G_{ik}^{\prime }\right)+\left(\begin{array}{c} N\\ 4 \end{array}\right)(\mu_{13}EG_{ij}^{\prime 2}G_{jk}^{\prime }G_{kl}^{\prime }+\\ \mu_{14}EG_{ij}^{\prime }G_{jk}^{\prime 2}G_{kl}^{\prime }+\mu_{15}EG_{ij}^{\prime 2}G_{ik}^{\prime }G_{il}^{\prime }+\mu_{16}EG_{ij}^{\prime }G_{jk}^{\prime }G_{ik}^{\prime }G_{il}^{\prime }+\mu_{17}EG_{ij}^{\prime }G_{jk}^{\prime }G_{kl}^{\prime }G_{il}^{\prime }+\mu_{18}EG_{ij}^{\prime 2}G_{kl}^{\prime 2})+\\ \left(\begin{array}{c} N\\ 5 \end{array}\right)\left(\mu_{19}EG_{ij}^{\prime }G_{jk}^{\prime }G_{kl}^{\prime }G_{lm}^{\prime }+\mu_{20}EG_{ij}^{\prime }G_{ik}^{\prime }G_{il}^{\prime }G_{im}^{\prime }+\mu_{21}EG_{ij}^{\prime }G_{ik}^{\prime }G_{il}^{\prime }G_{lm}^{\prime }+\mu_{22}EG_{ij}^{\prime }G_{ik}^{\prime }G_{lm}^{\prime 2}\right)+\left(\begin{array}{c} N\\ 6 \end{array}\right)\mu_{23}EG_{ij}^{\prime }G_{ik}^{\prime }G_{lm}^{\prime }G_{ln}^{\prime } \end{array}$$

The constants $\left(\mu_{9},\cdots ,\mu_{23}\right)$ are dependent on D and are provided in Supplemental Data. Note that $Var_{0}\left(S_{M}|D^{\prime }\right)\sim O\left(N^{3}\right),E_{0}\left(S_{M}^{3}|D^{\prime }\right)\sim O\left(N^{4}\right)$; thus, $\gamma \sim O\left(1/\sqrt{N}\right)$. Similarly, we can prove κ~O(1/N).

## Appendix E. Improve *p*-Value Approximations by Adjusting for Skewness and Kurtosis

We assume that $E_{0}Z=0$, $Var_{0}Z=1$, $\gamma =E_{0}Z^{3}$ and $\kappa =E_{0}Z^{4}-3$ under the original probability measure $P_{0}$. The tail probability $P_{0}(Z>b)$ for a large value of b is sensitive to the non-normality of Z, characterized by γ and κ. We define a new probability measure by embedding to the exponential probability density

(A10) $$\;dP_{\xi }=\exp\left(\xi Z-\phi \left(\xi \right)\right)DP_{0}$$

where $\phi \left(\xi \right)=\log E_{0}\exp\left(\xi Z\right)\;$is the log moment generating function. Note that $\gamma =\phi^{\prime \prime \prime }\left(0\right)$ and $\kappa =\phi^{\prime \prime \prime \prime }\left(0\right)$. Because $E_{0}\left(Z\right)=0$ and $Var_{0}\left(Z\right)=1$, Taylor’s expansion leads to $\phi \left(\xi \right)\approx \xi^{2}/2+\gamma \xi^{3}/6+\kappa \xi^{4}/24$. Under $P_{\xi }$, we have

(A11) $$E_{\xi }Z=\int ZdP_{\xi }=\phi^{\prime }\left(\xi \right)\approx \xi +\frac{\gamma }{2}\xi^{2}+\frac{\kappa }{6}\xi^{3}\;\;\;\;\;\;\;\;\;\;\;\;\;\;\;\;\;\;\;\;\;\;\;\;\;\;\;\;\;\;\;\;\;\;\;$$

and

(A12) $$Var_{\xi }Z=\phi^{\prime \prime }\left(\xi \right)\approx 1+\gamma \xi +\frac{\kappa }{2}\xi^{2}$$

We choose ξ such that $E_{\xi }Z\approx b$ by numerically solving an equation

(A13) $$\xi +\frac{\gamma }{2}\xi^{2}+\frac{\kappa }{6}\xi^{3}=b\;\;\;\;\;\;\;\;\;\;\;\;\;\;\;\;\;\;\;\;\;\;\;\;\;\;\;\;\;\;\;\;\;\;\;\;\;\;\;\;\;\;\;\;\;\;\;\;\;\;\;\;\;\;\;\;$$

Under the probability measure $P_{\xi }$, $Z\sim N\left(b,\sigma^{2}\right)$ approximately with $\sigma^{2}=1+\gamma \xi +k\xi^{2}/2$ in (A12). By the likelihood ratio identity and (A10), we have

(A14) $$\;\;P_{0}\left(Z>b\right)=E_{0}I_{Z>b}=E_{\xi }\frac{dP_{0}}{dP_{\xi }}I_{Z>b}=E_{\xi }e^{\phi \left(\xi \right)-\xi Z}I_{Z>b}=e^{\phi \left(\xi \right)}E_{\xi }e^{-\xi Z}I_{Z>b}\;\;\;\;\;\;$$

Note that $e^{-\xi Z}$ decays very fast when Z increases. Thus, the integral $E_{\xi }e^{-\xi Z}I_{Z>b}$ does not heavily depend on the tail distribution of Z. Assuming $Z\sim N\left(b,\sigma^{2}\right)$ under $P_{\xi }$, we can verify that

(A15) $$E_{\xi }e^{-\xi Z}I_{Z>b}=e^{-b\xi +\frac{\sigma^{2}\xi^{2}}{2}}\Phi \left(-\sigma \xi \right)\;\;\;\;\;\;\;\;\;\;\;\;\;\;\;\;\;\;\;\;\;\;\;\;\;\;\;\;\;\;\;\;\;\;\;\;\;\;\;\;\;\;\;\;\;\;\;$$

Combining (A14) and (A15) gives $P_{0}\left(Z>b\right)\approx \exp\left(\phi \left(\xi \right)-b\xi +\sigma^{2}\xi^{2}/2\right)\;\Phi \left(-\sigma \xi \right),$ which is further approximated as

$$\;P_{0}\left(Z>b\right)\approx \exp\left(-b\xi +\frac{1+\sigma^{2}}{2}\xi^{2}+\frac{\gamma }{6}\xi^{3}+\frac{\kappa }{24}\xi^{4}\right)\Phi \left(-\sigma \xi \right),$$

because $\phi \left(\xi \right)\approx \xi^{2}/2+\gamma \xi^{3}/6+\kappa \xi^{4}/24$ based on the Taylor expansion. This proves (9). If we correct skewness but assume kurtosis κ=0, then $\phi \left(\xi \right)\approx \xi^{2}/2+\gamma \xi^{3}/6$. We recalculate ξ by setting κ=0 in (A13) to derive $\xi =\left(\sqrt{1+2\gamma b}-1\right)/\gamma$. This proves (10).
