# Supplementary material for: MicrobiomeGWAS: A Tool for Identifying Host Genetic Variants Associated with Microbiome Composition
Source: Genes (Basel). 2022 Jul 9;13(7):1224. doi: 10.3390/genes13071224 (PMC9317577; doi:10.3390/genes13071224)
Supplement: Supplementary file 1 [file genes-13-01224-s001.zip › genes-1778170-supplementary.pdf]

| Unweighted UniFrac | | $Z_M$ | | | $Z_I$ | | | $Q$ | | |
|---|---|---|---|---|---|---|---|---|---|---|
| $MAF = 0.2$ | N | $\alpha$ =$10^{-3}$ | $10^{-5}$ | $10^{-7}$ | $10^{-3}$ | $10^{-5}$ | $10^{-7}$ | $10^{-3}$ | $10^{-5}$ | $10^{-7}$ |
| asymptotic approximation | 100 | 3.3 | 20.2 | 159.8 | 2.7 | 11.9 | 65.7 | 4.0 | 26.2 | 240.4 |
| | 200 | 2.6 | 11.6 | 68.5 | 2.2 | 7.6 | 33.1 | 3.0 | 14.5 | 89.8 |
| | 500 | 1.8 | 4.9 | 15.5 | 1.6 | 3.4 | 7.8 | 2.0 | 5.3 | 17.9 |
| | 1000 | 1.6 | 3.2 | 8.7 | 1.4 | 2.5 | 6.4 | 1.6 | 3.3 | 9.6 |
| adjusted for skewness and kurtosis | 100 | 1.0 | 1.2 | 0.7 | 1.0 | 1.0 | 1.0 | 1.0 | 1.3 | 1.5 |
| | 200 | 1.0 | 1.1 | 1.8 | 1.0 | 1.0 | 1.0 | 1.1 | 1.1 | 1.9 |
| | 500 | 1.0 | 1.0 | 1.4 | 1.0 | 0.9 | 0.7 | 0.9 | 0.9 | 0.7 |
| | 1000 | 1.0 | 0.9 | 1.2 | 1.0 | 0.9 | 1.0 | 0.9 | 1.0 | 0.6 |
| $MAF = 0.5$ | N | $\alpha$ =$10^{-3}$ | $10^{-5}$ | $10^{-7}$ | $10^{-3}$ | $10^{-5}$ | $10^{-7}$ | $10^{-3}$ | $10^{-5}$ | $10^{-7}$ |
| asymptotic approximation | 100 | 1.7 | 4.2 | 11.3 | 2.7 | 12.2 | 70.2 | 2.6 | 9.7 | 48.4 |
| | 200 | 1.4 | 2.8 | 5.7 | 2.2 | 7.1 | 30.0 | 2.0 | 5.7 | 19.8 |
| | 500 | 1.3 | 1.9 | 4.0 | 1.6 | 3.4 | 9.2 | 1.6 | 2.9 | 6.8 |
| | 1000 | 1.2 | 1.6 | 2.6 | 1.4 | 2.6 | 4.9 | 1.4 | 2.2 | 3.9 |
| adjusted for skewness and kurtosis | 100 | 1.0 | 0.9 | 0.8 | 1.0 | 1.1 | 1.5 | 1.0 | 1.0 | 0.9 |
| | 200 | 1.0 | 1.0 | 1.0 | 1.0 | 1.0 | 0.6 | 1.0 | 1.0 | 0.8 |
| | 500 | 1.0 | 1.0 | 0.9 | 1.0 | 1.0 | 1.0 | 1.0 | 1.0 | 0.8 |
| | 1000 | 1.0 | 1.0 | 1.4 | 1.0 | 1.0 | 0.4 | 1.0 | 1.0 | 0.9 |

| Weighted UniFrac | | $Z_M$ | | | $Z_I$ | | | $Q$ | | |
|---|---|---|---|---|---|---|---|---|---|---|
| $MAF = 0.2$ | N | $\alpha$ =$10^{-3}$ | $10^{-5}$ | $10^{-7}$ | $10^{-3}$ | $10^{-5}$ | $10^{-7}$ | $10^{-3}$ | $10^{-5}$ | $10^{-7}$ |
| asymptotic approximation | 100 | 5.5 | 51.6 | 610.0 | 4.7 | 36.1 | 342.8 | 7.3 | 80.9 | 1147.9 |
| | 200 | 3.7 | 23.0 | 187.3 | 3.1 | 15.8 | 105.5 | 4.6 | 32.9 | 316.7 |
| | 500 | 2.4 | 9.4 | 45.2 | 2.1 | 6.7 | 25.5 | 2.8 | 11.9 | 64.1 |
| | 1000 | 2.0 | 5.7 | 21.3 | 1.8 | 4.4 | 14.0 | 2.2 | 6.9 | 28.5 |
| adjusted for skewness and kurtosis | 100 | 1.0 | 1.2 | 0.7 | 1.0 | 1.1 | 0.6 | 1.0 | 1.5 | 2.0 |
| | 200 | 1.0 | 1.1 | 1.0 | 1.0 | 1.1 | 0.7 | 0.9 | 1.3 | 1.8 |
| | 500 | 1.0 | 1.1 | 1.3 | 1.0 | 1.0 | 0.9 | 0.9 | 1.0 | 1.7 |
| | 1000 | 1.0 | 1.0 | 1.2 | 1.0 | 1.0 | 0.8 | 0.9 | 1.0 | 1.1 |
| $MAF = 0.5$ | N | $\alpha$ =$10^{-3}$ | $10^{-5}$ | $10^{-7}$ | $10^{-3}$ | $10^{-5}$ | $10^{-7}$ | $10^{-3}$ | $10^{-5}$ | $10^{-7}$ |
| asymptotic approximation | 100 | 1.8 | 4.2 | 10.8 | 4.0 | 25.7 | 207.1 | 3.6 | 19.2 | 142.1 |
| | 200 | 1.5 | 2.8 | 6.9 | 2.8 | 12.0 | 67.3 | 2.5 | 9.1 | 41.6 |
| | 500 | 1.3 | 1.9 | 3.4 | 2.0 | 5.6 | 22.2 | 1.8 | 4.3 | 13.9 |
| | 1000 | 1.2 | 1.5 | 2.4 | 1.7 | 3.9 | 11.2 | 1.6 | 3.0 | 7.0 |
| adjusted for skewness and kurtosis | 100 | 1.0 | 1.0 | 0.6 | 1.0 | 1.0 | 0.5 | 1.0 | 1.1 | 0.7 |
| | 200 | 1.0 | 1.0 | 1.0 | 1.0 | 1.0 | 0.6 | 1.0 | 1.1 | 0.4 |
| | 500 | 1.0 | 0.9 | 1.0 | 1.0 | 1.0 | 0.8 | 1.0 | 1.1 | 1.2 |
| | 1000 | 1.0 | 1.0 | 0.7 | 1.0 | 1.0 | 1.0 | 1.0 | 1.0 | 1.3 |

**Table S1**: Type-I error rates estimated based on $10^8$ simulations. Minor allele frequency = 20% and 50%. Simulations were based on the weighted and the unweighted UniFrac distance matrices of the gut microbiome data from the American Gut Project. Reported are the type-I error inflation factor. A value greater than 1 indicates an inflated type-I error.

| | | | unweighted UniFrac | | | weighted UniFrac | | |
|---|---|---|---|---|---|---|---|---|
| SNP | locus | Annotated gene | $P_M$ | $P_I$ | $P_{Joint}$ | $P_M$ | $P_I$ | $P_{Joint}$ |
| rs2036534 | 15q25.1 | *CHRNA3/4/5* | 0.425 | 0.307 | 0.610 | 0.167 | 0.039 | 0.111 |
| rs1051730 | | | 0.020 | 0.174 | 0.053 | 0.401 | 0.426 | 0.675 |
| rs2736100 | 5p15.33 | *TERT* | 0.089 | 0.252 | 0.201 | 0.267 | 0.257 | 0.435 |
| rs401681 | | *CLPTM1L* | 0.056 | 0.898 | 0.047 | 0.005 | 0.379 | 0.013 |
| rs6489769 | 12p13.3 | *RAD52* | 0.584 | 0.403 | 0.656 | 0.598 | 0.632 | 0.794 |
| rs1333040 | 9p21.3 | *CDKN2A/CDKN2B* | 0.249 | 0.614 | 0.405 | 0.224 | 0.437 | 0.453 |

**Table S2**: Association P-values between lung cancer risk SNPs and microbiome composition in the EAGLE data. $P_M$: P-values for testing main effects. $P_I$: P-values for testing SNP/smoking interactions. $P_{Joint}$: P-value for jointly testing main and interaction effects.

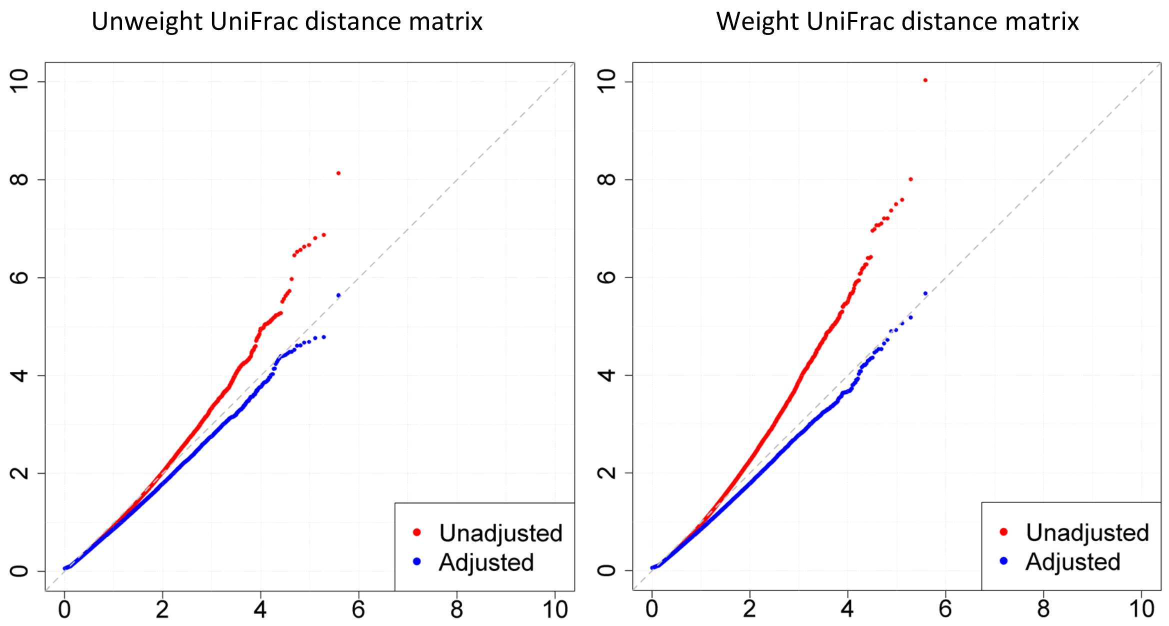


**Figure S1**: Quantile-quantile (QQ) plot for association P-values testing the joint effects (main effect and SNP by smoking interaction) using the unweighted UniFrac distance matrices. "Adjusted": P-values were corrected for skewness and kurtosis. "Unadjusted": P-values were approximated based on the asymptotic distribution $N(0,1)$. The left (right) panel was based on the analysis using the unweighted (weighted) UniFrac distance matrix.

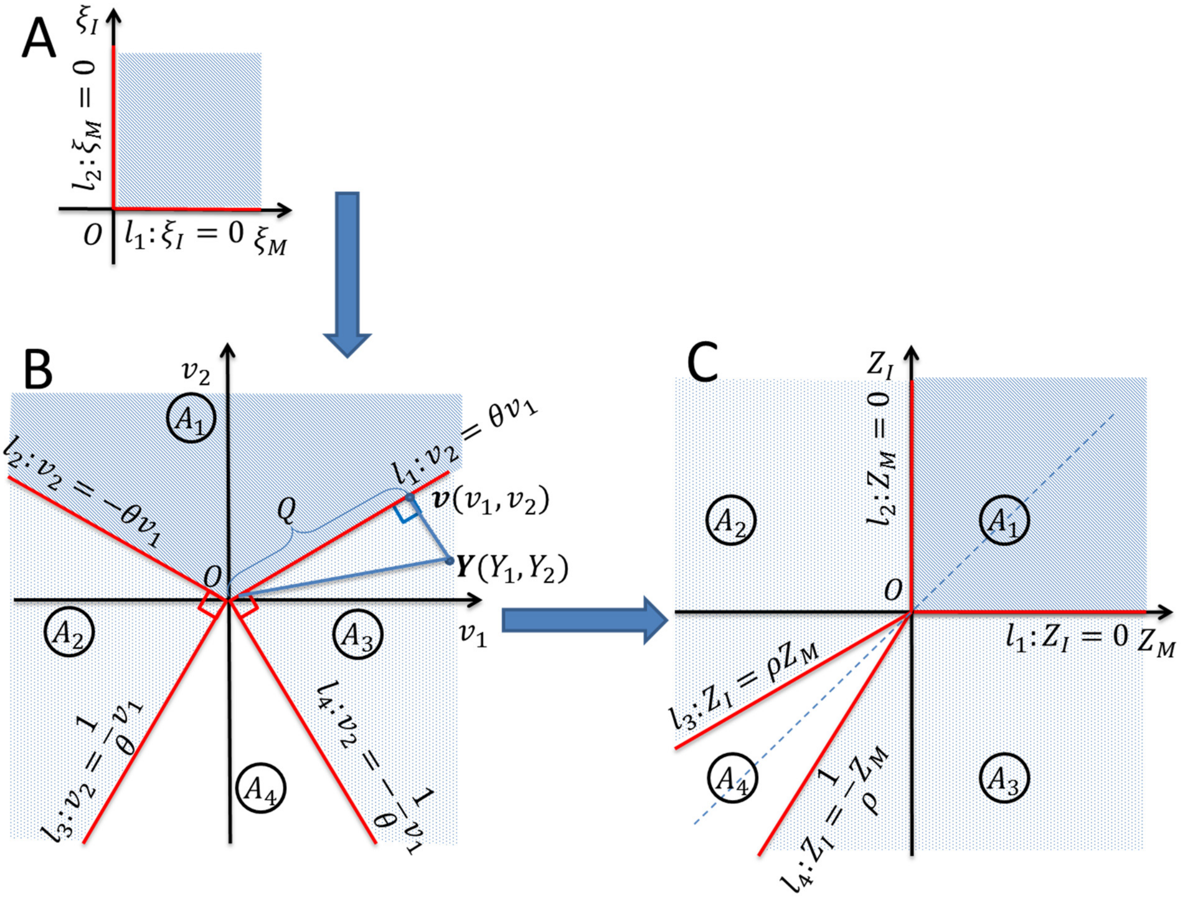


**Figure S2**: Derivation of the likelihood ratio statistic $\boldsymbol{Q}$ in (7) and (8). (A) The original parameter space (shadow area). (B) The transformed parameter space. The two lines $\boldsymbol{l_1}$ and $\boldsymbol{l_2}$ characterizing the boundaries in (A) are transformed to $\boldsymbol{l_1}$ and $\boldsymbol{l_2}$ in (B). When $(\boldsymbol{Y_1}, \boldsymbol{Y_2}) \in \boldsymbol{A_1}$, Q is maximized when $\boldsymbol{v_1} = \boldsymbol{Y_1}$ and $\boldsymbol{v_2} = \boldsymbol{Y_2}$, which leads to $\boldsymbol{Q} = \boldsymbol{Y^T Y}$. When $(\boldsymbol{Y_1}, \boldsymbol{Y_2}) \in \boldsymbol{A_4}$, Q is maximized when $\boldsymbol{v_1} = \boldsymbol{0}$ and $\boldsymbol{v_2} = \boldsymbol{0}$, which leads to $\boldsymbol{Q} = \boldsymbol{0}$. When $(\boldsymbol{Y_1}, \boldsymbol{Y_2}) \in \boldsymbol{A_3}$, Q is maximized when $(\boldsymbol{v_1}, \boldsymbol{v_2})$ is the projection of $(\boldsymbol{Y_1}, \boldsymbol{Y_2})$ onto the boundary represented as $\boldsymbol{l_1}$ in B, which leads to $\boldsymbol{Q} = (\boldsymbol{Y_2} + \boldsymbol{Y_1}/\boldsymbol{\theta})^2/(\boldsymbol{1} + \boldsymbol{\theta}^{-2})$. Q can be similarly derived when $(\boldsymbol{Y_1}, \boldsymbol{Y_2}) \in \boldsymbol{A_2}$. (C). We perform an inverse transformation using $\boldsymbol{\Sigma}^{1/2}$ to the original parameter space. The four lines characterizing the four parts are now represented in C.

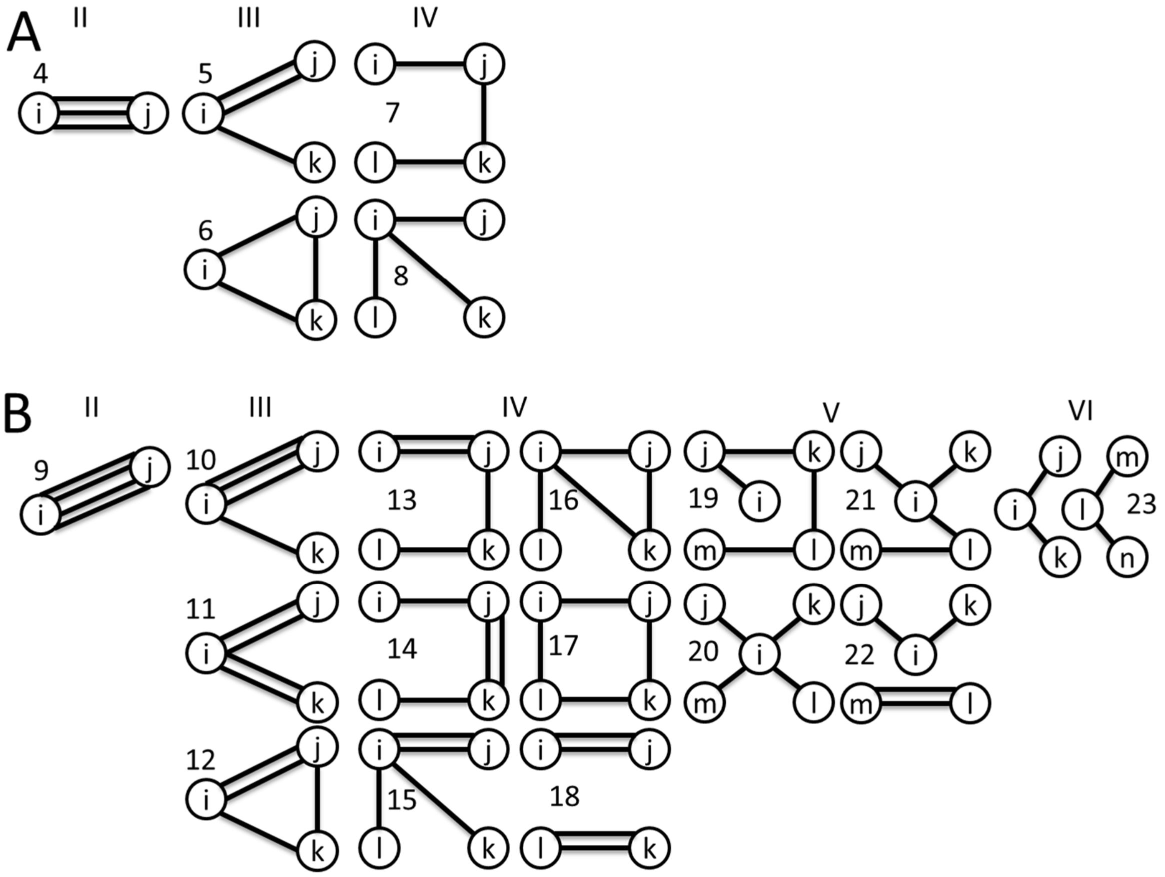


**Figure S3**: (A) All combinations of $(\boldsymbol{i}, \boldsymbol{j}, \boldsymbol{m}, \boldsymbol{n}, \boldsymbol{s}, \boldsymbol{t})$ with $\boldsymbol{EG'_{ij}G'_{mn}G'_{st}} \neq \mathbf{0}$, where $\boldsymbol{G'_{ij}} = \boldsymbol{G_{ij}} - \boldsymbol{EG_{ij}}$. (B) All 15 combinations of $(\boldsymbol{i}, \boldsymbol{j}, \boldsymbol{m}, \boldsymbol{n}, \boldsymbol{s}, \boldsymbol{t}, \boldsymbol{x}, \boldsymbol{y})$ with $\boldsymbol{EG'_{ij}G'_{mn}G'_{st}G'_{xy}} \neq \mathbf{0}$. For example, The figure labeled as "4" represents $\boldsymbol{EG'^{3}_{ij}} \neq \mathbf{0}$. The figure labeled as "5" represents $\boldsymbol{EG'^{2}_{ij}G'_{ik}} \neq \mathbf{0}$, the figure labeled as "10" represents $\boldsymbol{EG'^{3}_{ij}G'_{ik}} \neq \mathbf{0}$, The figure labeled as "18" represents $\boldsymbol{EG'^{2}_{ij}G'^{2}_{kl}} \neq \mathbf{0}$. All detailed calculations are in the next pages.

The expectation of the corresponding product of $G_{ij} = |g_i - g_j|$ could be calculated based on the distribution of $g_i$. Denote $p_t = \mathrm{P}(g_i = t), t = 0,1,2$, we can write the formula directly from the definition. Here we list the formulas of the combination in **Figure S3A**.

$$\begin{cases} \mathrm{E}(G_{ij}^3) = \sum_{i,j,k\in\{0,1,2\}} p_i p_j p_k |i-j|^3 = 2p_0p_1 + 2p_1p_2 + 16p_0p_2 \\ \mathrm{E}(G_{ij}^2 G_{ik}) = \sum_{i,j,k\in\{0,1,2\}} p_i p_j p_k |i-j|^2|i-k| = p_1(1-p_1) + 4p_0p_2(2+p_1) \\ \mathrm{E}(G_{ij}G_{jk}G_{ik}) = \sum_{i,j,k\in\{0,1,2\}} p_i p_j p_k |i-j||j-k||i-k| = 12p_0p_1p_2 \\ \mathrm{E}(G_{ij}G_{jk}G_{kl}) = \sum_{i,j,k,l\in\{0,1,2\}} p_i p_j p_k p_l |i-j||j-k||k-l| = 2p_1^2 + 2(p_1^2 - 4p_0p_2)(p_1^2 - 2p_1 - 2p_0p_2) \\ \mathrm{E}(G_{ij}G_{ik}G_{il}) = \sum_{i,j,k,l\in\{0,1,2\}} p_i p_j p_k p_l |i-j||i-k||i-l| = p_0(p_1+2p_2)^3 + p_1(p_0+p_2)^3 + p_2(2p_0+p_1)^3 \end{cases} \quad \text{(S1)}$$

The formulas of the combination in **Figure S3B** is a little complicated, we just list the summation.

$$\begin{cases} \mathrm{E}(G_{ij}^4) = \sum_{i,j\in\{0,1,2\}} p_i p_j |i-j|^4 \\ \mathrm{E}(G_{ij}^3 G_{ik}) = \sum_{i,j,k\in\{0,1,2\}} p_i p_j p_k |i-j|^3|i-k| \\ \mathrm{E}(G_{ij}^2 G_{ik}^2) = \sum_{i,j,k\in\{0,1,2\}} p_i p_j p_k |i-j|^2|i-k|^2 \\ \mathrm{E}(G_{ij}^2 G_{jk} G_{ik}) = \sum_{i,j,k\in\{0,1,2\}} p_i p_j p_k |i-j|^2|j-k||i-k| \\ \mathrm{E}(G_{ij}^2 G_{jk} G_{kl}) = \sum_{i,j,k,l\in\{0,1,2\}} p_i p_j p_k p_l |i-j|^2|j-k||k-l| \\ \mathrm{E}(G_{ij} G_{jk}^2 G_{kl}) = \sum_{i,j,k,l\in\{0,1,2\}} p_i p_j p_k p_l |i-j||j-k|^2|k-l| \\ \mathrm{E}(G_{ij}^2 G_{ik} G_{il}) = \sum_{i,j,k,l\in\{0,1,2\}} p_i p_j p_k p_l |i-j|^2|i-k||i-l| \\ \mathrm{E}(G_{ij}G_{jk}G_{ik}G_{il}) = \sum_{i,j,k,l\in\{0,1,2\}} p_i p_j p_k p_l |i-j||j-k||i-k||i-l| \\ \mathrm{E}(G_{ij}G_{jk}G_{kl}G_{il}) = \sum_{i,j,k,l\in\{0,1,2\}} p_i p_j p_k p_l |i-j||j-k||k-l||i-l| \\ \mathrm{E}(G_{ij}^2 G_{kl}^2) = \sum_{i,j,k,l\in\{0,1,2\}} p_i p_j p_k p_l |i-j|^2|k-l|^2 = \mathrm{E}(G_{ij}^2)^2 \\ \mathrm{E}(G_{ij}G_{jk}G_{kl}G_{lm}) = \sum_{i,j,k,l,m\in\{0,1,2\}} p_i p_j p_k p_l p_m |i-j||j-k||k-l||l-m| \\ \mathrm{E}(G_{ij}G_{ik}G_{il}G_{im}) = \sum_{i,j,k,l,m\in\{0,1,2\}} p_i p_j p_k p_l p_m |i-j||i-k||i-l||i-m| \\ \mathrm{E}(G_{ij}G_{ik}G_{il}G_{lm}) = \sum_{i,j,k,l,m\in\{0,1,2\}} p_i p_j p_k p_l p_m |i-j||i-k||i-l||l-m| \\ \mathrm{E}(G_{ij}G_{ik}G_{lm}^2) = \sum_{i,j,k,l,m\in\{0,1,2\}} p_i p_j p_k p_l p_m |i-j||i-k||l-m|^2 = \mathrm{E}(G_{ij}G_{ik})\mathrm{E}(G_{ij}^2) \\ \mathrm{E}(G_{ij}G_{ik}G_{lm}G_{ln}) = \sum_{i,j,k,l,m,n\in\{0,1,2\}} p_i p_j p_k p_l p_m p_n |i-j||i-k||l-m||l-n| = \mathrm{E}(G_{ij}G_{ik})^2 \end{cases} \quad \text{(S2)}$$

Based on the definition that $\bar{G}_{ij} = G_{ij} - \mathrm{E}(G_{ij})$, the expectation of the product of $\bar{G}_{ij}$ could be calculated from the following equations:

$$
\begin{cases}
\mathrm{E}(\bar{G}_{ij}^3) = \mathrm{E}(G_{ij}^3) - 3\mathrm{E}(G_{ij}^2)\mathrm{E}(G_{ij}) + 2\mathrm{E}(G_{ij})^3 \\
\mathrm{E}(\bar{G}_{ij}^2\bar{G}_{ik}) = \mathrm{E}(G_{ij}^2G_{ik}) - \mathrm{E}(G_{ij}^2)\mathrm{E}(G_{ij}) - 2\mathrm{E}(G_{ij}G_{ik})\mathrm{E}(G_{ij}) + 2\mathrm{E}(G_{ij})^3 \\
\mathrm{E}(\bar{G}_{ij}\bar{G}_{jk}\bar{G}_{ik}) = \mathrm{E}(G_{ij}G_{jk}G_{ik}) - 3\mathrm{E}(G_{ij}G_{ik})\mathrm{E}(G_{ij}) + 2\mathrm{E}(G_{ij})^3 \\
\mathrm{E}(\bar{G}_{ij}\bar{G}_{jk}\bar{G}_{kl}) = \mathrm{E}(G_{ij}G_{jk}G_{kl}) - 2\mathrm{E}(G_{ij}G_{ik})\mathrm{E}(G_{ij}) + \mathrm{E}(G_{ij})^3 \\
\mathrm{E}(\bar{G}_{ij}\bar{G}_{ik}\bar{G}_{il}) = \mathrm{E}(G_{ij}G_{ik}G_{il}) - 3\mathrm{E}(G_{ij}G_{ik})\mathrm{E}(G_{ij}) + 2\mathrm{E}(G_{ij})^3 \\
\mathrm{E}(\bar{G}_{ij}^4) = \mathrm{E}(G_{ij}^4) - 4\mathrm{E}(G_{ij}^3)\mathrm{E}(G_{ij}) + 6\mathrm{E}(G_{ij}^2)\mathrm{E}(G_{ij})^2 - 3\mathrm{E}(G_{ij})^4 \\
\mathrm{E}(\bar{G}_{ij}^3\bar{G}_{ik}) = \mathrm{E}(G_{ij}^3G_{ik}) - \begin{pmatrix} 3\mathrm{E}(G_{ij}^2G_{ik}) \\ -\mathrm{E}(G_{ij}^3) \end{pmatrix}\mathrm{E}(G_{ij}) + 3\begin{pmatrix} \mathrm{E}(G_{ij}G_{ik}) \\ +\mathrm{E}(G_{ij}^2) \end{pmatrix}\mathrm{E}(G_{ij})^2 - 3\mathrm{E}(G_{ij})^4 \\
\mathrm{E}(\bar{G}_{ij}^2\bar{G}_{ik}^2) = \mathrm{E}(G_{ij}^2G_{ik}^2) - 4\mathrm{E}(G_{ij}^2G_{ik})\mathrm{E}(G_{ij}) + \begin{pmatrix} 4\mathrm{E}(G_{ij}G_{ik}) \\ +2\mathrm{E}(G_{ij}^2) \end{pmatrix}\mathrm{E}(G_{ij})^2 - 3\mathrm{E}(G_{ij})^4 \\
\mathrm{E}(\bar{G}_{ij}^2\bar{G}_{jk}\bar{G}_{ik}) = \mathrm{E}(G_{ij}^2G_{jk}G_{ik}) - 2\begin{pmatrix} \mathrm{E}(G_{ij}G_{jk}G_{ik}) \\ +\mathrm{E}(G_{ij}^2G_{ik}) \end{pmatrix}\mathrm{E}(G_{ij}) + \begin{pmatrix} 5\mathrm{E}(G_{ij}G_{ik}) \\ +\mathrm{E}(G_{ij}^2) \end{pmatrix}\mathrm{E}(G_{ij})^2 - 3\mathrm{E}(G_{ij})^4 \\
\mathrm{E}(\bar{G}_{ij}^2\bar{G}_{jk}\bar{G}_{kl}) = \mathrm{E}(G_{ij}^2G_{jk}G_{kl}) - \begin{pmatrix} 2\mathrm{E}(G_{ij}G_{jk}G_{kl}) \\ +\mathrm{E}(G_{ij}^2G_{ik}) \end{pmatrix}\mathrm{E}(G_{ij}) + 3\mathrm{E}(G_{ij}G_{ik})\mathrm{E}(G_{ij})^2 - \mathrm{E}(G_{ij})^4 \\
\mathrm{E}(\bar{G}_{ij}\bar{G}_{jk}^2\bar{G}_{kl}) = \mathrm{E}(G_{ij}G_{jk}^2G_{kl}) - 2\begin{pmatrix} \mathrm{E}(G_{ij}G_{jk}G_{kl}) \\ +\mathrm{E}(G_{ij}^2G_{ik}) \end{pmatrix}\mathrm{E}(G_{ij}) + \begin{pmatrix} 4\mathrm{E}(G_{ij}G_{ik}) \\ +\mathrm{E}(G_{ij}^2) \end{pmatrix}\mathrm{E}(G_{ij})^2 - 2\mathrm{E}(G_{ij})^4 \\
\mathrm{E}(\bar{G}_{ij}^2\bar{G}_{ik}\bar{G}_{il}) = \mathrm{E}(G_{ij}^2G_{ik}G_{il}) - 2\begin{pmatrix} \mathrm{E}(G_{ij}G_{jk}G_{kl}) \\ +\mathrm{E}(G_{ij}^2G_{ik}) \end{pmatrix}\mathrm{E}(G_{ij}) + \begin{pmatrix} 5\mathrm{E}(G_{ij}G_{ik}) \\ +\mathrm{E}(G_{ij}^2) \end{pmatrix}\mathrm{E}(G_{ij})^2 - 3\mathrm{E}(G_{ij})^4 \\
\mathrm{E}(\bar{G}_{ij}\bar{G}_{jk}\bar{G}_{ik}\bar{G}_{il}) = \mathrm{E}(G_{ij}G_{jk}G_{ik}G_{il}) - \begin{pmatrix} \mathrm{E}(G_{ij}G_{ik}G_{il}) \\ +\mathrm{E}(G_{ij}G_{jk}G_{ik}) \\ +2\mathrm{E}(G_{ij}G_{jk}G_{kl}) \end{pmatrix}\mathrm{E}(G_{ij}) + 5\mathrm{E}(G_{ij}G_{ik})\mathrm{E}(G_{ij})^2 - 2\mathrm{E}(G_{ij})^4 \\
\mathrm{E}(\bar{G}_{ij}\bar{G}_{jk}\bar{G}_{kl}\bar{G}_{il}) = \mathrm{E}(G_{ij}G_{jk}G_{kl}G_{il}) - 4\mathrm{E}(G_{ij}G_{jk}G_{kl})\mathrm{E}(G_{ij}) + 4\mathrm{E}(G_{ij}G_{ik})\mathrm{E}(G_{ij})^2 - \mathrm{E}(G_{ij})^4 \\
\mathrm{E}(\bar{G}_{ij}^2\bar{G}_{kl}^2) = \mathrm{E}(\bar{G}_{ij}^2)^2 = \left(\mathrm{E}(G_{ij}^2) - \mathrm{E}(G_{ij})^2\right)^2 \\
\mathrm{E}(\bar{G}_{ij}\bar{G}_{jk}\bar{G}_{kl}\bar{G}_{lm}) = \mathrm{E}(G_{ij}G_{jk}G_{kl}G_{lm}) - 2\mathrm{E}(G_{ij}G_{jk}G_{kl})\mathrm{E}(G_{ij}) + \mathrm{E}(G_{ij}G_{ik})\mathrm{E}(G_{ij})^2 \\
\mathrm{E}(\bar{G}_{ij}\bar{G}_{ik}\bar{G}_{il}\bar{G}_{im}) = \mathrm{E}(G_{ij}G_{ik}G_{il}G_{im}) - 4\mathrm{E}(G_{ij}G_{ik}G_{il})\mathrm{E}(G_{ij}) + 6\mathrm{E}(G_{ij}G_{ik})\mathrm{E}(G_{ij})^2 - 3\mathrm{E}(G_{ij})^4 \\
\mathrm{E}(\bar{G}_{ij}\bar{G}_{ik}\bar{G}_{il}\bar{G}_{lm}) = \mathrm{E}(G_{ij}G_{ik}G_{il}G_{lm}) - \begin{pmatrix} 2\mathrm{E}(G_{ij}G_{jk}G_{kl}) \\ +\mathrm{E}(G_{ij}G_{ik}G_{il}) \end{pmatrix}\mathrm{E}(G_{ij}) + 3\mathrm{E}(G_{ij}G_{ik})\mathrm{E}(G_{ij})^2 - \mathrm{E}(G_{ij})^4 \\
\mathrm{E}(\bar{G}_{ij}\bar{G}_{ik}\bar{G}_{lm}^2) = \mathrm{E}(\bar{G}_{ij}\bar{G}_{ik})\mathrm{E}(\bar{G}_{ij}^2) = \left(\mathrm{E}(G_{ij}G_{ik}) - \mathrm{E}(G_{ij})^2\right)\left(\mathrm{E}(G_{ij}^2) - \mathrm{E}(G_{ij})^2\right) \\
\mathrm{E}(\bar{G}_{ij}\bar{G}_{ik}\bar{G}_{lm}\bar{G}_{ln}) = \mathrm{E}(\bar{G}_{ij}\bar{G}_{ik})^2 = \left(\mathrm{E}(G_{ij}G_{ik}) - \mathrm{E}(G_{ij})^2\right)^2
\end{cases}
\quad \text{(S3)}
$$

$\mu_i, i = 4,5, \dots, 23$ in **Appendix D** could be calculated by averaging the products of all possible $\bar{d}_{ij}$ combinations in **Figure S3** from the following equations:

$$
\begin{cases}
\mu_4 = \binom{N}{2}^{-1} \sum_{i<j<k} \bar{d}_{ij}^3 \\
\mu_5 = 18\binom{N}{3}^{-1} \sum_{i<j<k} \left(\bar{d}_{ij}^2(\bar{d}_{ik}+\bar{d}_{jk}) + \bar{d}_{jk}^2(\bar{d}_{ij}+\bar{d}_{ik}) + \bar{d}_{ik}^2(\bar{d}_{ij}+\bar{d}_{jk})\right)/6 \\
\mu_6 = 6\binom{N}{3}^{-1} \sum_{i<j<k} \bar{d}_{ij}\bar{d}_{jk}\bar{d}_{ik} \\
\mu_7 = 72\binom{N}{3}^{-1} \sum_{i<j<k} \begin{pmatrix} \bar{d}_{ij}\bar{d}_{kl}(\bar{d}_{ik}+\bar{d}_{il}+\bar{d}_{jk}+\bar{d}_{jl}) \\ +\bar{d}_{ik}\bar{d}_{jl}(\bar{d}_{ij}+\bar{d}_{il}+\bar{d}_{jk}+\bar{d}_{kl}) \\ +\bar{d}_{il}\bar{d}_{jk}(\bar{d}_{ij}+\bar{d}_{ik}+\bar{d}_{jl}+\bar{d}_{kl}) \end{pmatrix} \Big/ 12 \\
\mu_8 = 24\binom{N}{3}^{-1} \sum_{i<j<k} (\bar{d}_{ij}\bar{d}_{ik}\bar{d}_{il} + \bar{d}_{ij}\bar{d}_{jk}\bar{d}_{jl} + \bar{d}_{ik}\bar{d}_{jk}\bar{d}_{kl} + \bar{d}_{il}\bar{d}_{jl}\bar{d}_{kl})/4 \\
\mu_9 = \binom{N}{2}^{-1} \sum_{i<j} \bar{d}_{ij}^4 \\
\mu_{10} = 24\binom{N}{3}^{-1} \sum_{i<j} \left(\bar{d}_{ij}^3(\bar{d}_{ik}+\bar{d}_{jk}) + \bar{d}_{jk}^3(\bar{d}_{ij}+\bar{d}_{ik}) + \bar{d}_{ik}^3(\bar{d}_{ij}+\bar{d}_{jk})\right)/6 \\
\mu_{11} = 18\binom{N}{3}^{-1} \sum_{i<j<k} (\bar{d}_{ij}^2\bar{d}_{ik}^2 + \bar{d}_{ij}^2\bar{d}_{jk}^2 + \bar{d}_{ik}^2\bar{d}_{jk}^2)/3 \\
\mu_{12} = 36\binom{N}{3}^{-1} \sum_{i<j<k} \bar{d}_{ij}\bar{d}_{jk}\bar{d}_{ik}(\bar{d}_{ij}+\bar{d}_{jk}+\bar{d}_{ik})/3 \\
\mu_{13} = 288\binom{N}{4}^{-1} \sum_{i<j<k} \begin{pmatrix} \bar{d}_{ij}\bar{d}_{kl}(\bar{d}_{ij}+\bar{d}_{kl})(\bar{d}_{ik}+\bar{d}_{il}+\bar{d}_{jk}+\bar{d}_{jl}) \\ +\bar{d}_{ik}\bar{d}_{jl}(\bar{d}_{ik}+\bar{d}_{jl})(\bar{d}_{ij}+\bar{d}_{il}+\bar{d}_{jk}+\bar{d}_{kl}) \\ +\bar{d}_{il}\bar{d}_{jk}(\bar{d}_{il}+\bar{d}_{jk})(\bar{d}_{ij}+\bar{d}_{ik}+\bar{d}_{jl}+\bar{d}_{kl}) \end{pmatrix} \Big/ 24 \\
\mu_{14} = 144\binom{N}{4}^{-1} \sum_{i<j<k} \begin{pmatrix} \bar{d}_{ij}\bar{d}_{kl}(\bar{d}_{ik}^2+\bar{d}_{il}^2+\bar{d}_{jk}^2+\bar{d}_{jl}^2) \\ +\bar{d}_{ik}\bar{d}_{jl}(\bar{d}_{ij}^2+\bar{d}_{il}^2+\bar{d}_{jk}^2+\bar{d}_{kl}^2) \\ +\bar{d}_{il}\bar{d}_{jk}(\bar{d}_{ij}^2+\bar{d}_{ik}^2+\bar{d}_{jl}^2+\bar{d}_{kl}^2) \end{pmatrix} \Big/ 12 \\
\mu_{15} = 144\binom{N}{4}^{-1} \sum_{i<j<k} \begin{pmatrix} \bar{d}_{ij}\bar{d}_{ik}\bar{d}_{il}(\bar{d}_{ij}+\bar{d}_{ik}+\bar{d}_{il}) + \bar{d}_{ij}\bar{d}_{jk}\bar{d}_{jl}(\bar{d}_{ij}+\bar{d}_{jk}+\bar{d}_{jl}) \\ +\bar{d}_{ik}\bar{d}_{jk}\bar{d}_{kl}(\bar{d}_{ik}+\bar{d}_{jk}+\bar{d}_{kl}) + \bar{d}_{il}\bar{d}_{jl}\bar{d}_{kl}(\bar{d}_{il}+\bar{d}_{jl}+\bar{d}_{kl}) \end{pmatrix} /12 \\
\mu_{16} = 288\binom{N}{4}^{-1} \sum_{i<j<k} \begin{pmatrix} \bar{d}_{ij}\bar{d}_{jk}\bar{d}_{ik}(\bar{d}_{il}+\bar{d}_{jl}+\bar{d}_{kl}) + \bar{d}_{ij}\bar{d}_{jl}\bar{d}_{il}(\bar{d}_{ik}+\bar{d}_{jk}+\bar{d}_{kl}) \\ +\bar{d}_{ik}\bar{d}_{kl}\bar{d}_{il}(\bar{d}_{ij}+\bar{d}_{jk}+\bar{d}_{jl}) + \bar{d}_{jk}\bar{d}_{kl}\bar{d}_{jl}(\bar{d}_{ij}+\bar{d}_{ik}+\bar{d}_{il}) \end{pmatrix} /12 \\
\mu_{17} = 72\binom{N}{4}^{-1} \sum_{i<j<k} (\bar{d}_{ij}\bar{d}_{jk}\bar{d}_{kl}\bar{d}_{il} + \bar{d}_{ij}\bar{d}_{jl}\bar{d}_{kl}\bar{d}_{ik} + \bar{d}_{ik}\bar{d}_{jk}\bar{d}_{jl}\bar{d}_{il})/3 \\
\mu_{18} = 18\binom{N}{4}^{-1} \sum_{i<j<k} (\bar{d}_{ij}^2\bar{d}_{kl}^2 + \bar{d}_{ik}^2\bar{d}_{jl}^2 + \bar{d}_{il}^2\bar{d}_{jk}^2)/3 \\
\mu_{19} = 1440\binom{N}{5}^{-1} \sum_{i<j<k} (sum\ of\ all\ 60\ possible\ terms)/60 \\
\mu_{20} = 120\binom{N}{5}^{-1} \sum_{i<j<k} \begin{pmatrix} \bar{d}_{ij}\bar{d}_{ik}\bar{d}_{il}\bar{d}_{im} + \bar{d}_{ij}\bar{d}_{jk}\bar{d}_{jl}\bar{d}_{jm} + \bar{d}_{ik}\bar{d}_{jk}\bar{d}_{kl}\bar{d}_{km} \\ +\bar{d}_{il}\bar{d}_{jl}\bar{d}_{kl}\bar{d}_{lm} + \bar{d}_{im}\bar{d}_{jm}\bar{d}_{km}\bar{d}_{lm} \end{pmatrix} /5 \\
\mu_{21} = 1440\binom{N}{5}^{-1} \sum_{i<j<k} (sum\ of\ all\ 60\ possible\ terms)/60 \\
\mu_{22} = 360\binom{N}{5}^{-1} \sum_{i<j<k} (sum\ of\ all\ 30\ possible\ terms)/30 \\
\mu_{23} = 2160\binom{N}{6}^{-1} \sum_{i<j<k} (sum\ of\ all\ 90\ possible\ terms)/90
\end{cases}
\quad \text{(S4)}
$$

Calculation of $\mathrm{Cov}(G_{ij}, \Delta_{ij})$ and $\mathrm{Cov}(G_{ij}, \Delta_{ik})$.

| Probabilities | $E_i = 0$ | $E_i = 1$ | Sum |
|---|---|---|---|
| $g_i = 0$ | $p_{00}$ | $p_{01}$ | $p_{0\cdot}$ |

| | | | |
|---|---|---|---|
| $g_i = 1$ | $p_{10}$ | $p_{11}$ | $p_{1\cdot}$ |
| $g_i = 2$ | $p_{20}$ | $p_{21}$ | $p_{2\cdot}$ |
| Sum | $p_{\cdot 0}$ | $p_{\cdot 1}$ | 1 |

This above table lists the joint distribution of $(g, E)$. Based on this table, we have

$$\begin{cases} \mathrm{E}(G_{ij}) = \sum_{i,j\in\{0,1,2\}} p_{i\cdot}p_{j\cdot}|i-j| = 2p_{0\cdot}p_{1\cdot} + 2p_{1\cdot}p_{2\cdot} + 4p_{0\cdot}p_{2\cdot} \\ \mathrm{E}(\Delta_{ij}) = \sum_{i,j\in\{0,1,2\}} \sum_{a,b\in\{0,1\}} p_{ia}p_{jb}|ia-jb| \\ \quad = 2p_{11}(1-p_{11}) + 4p_{21}(1-p_{11}-p_{21}) \\ \mathrm{E}(G_{ij}\Delta_{ij}) = \sum_{i,j\in\{0,1,2\}} \sum_{a,b\in\{0,1\}} p_{ia}p_{jb}|i-j||ia-jb| \\ \quad = 2p_{11}(p_{0\cdot} + p_{2\cdot}) + 4p_{21}p_{10} + 8p_{21}p_{0\cdot} \\ \mathrm{E}(G_{ij}\Delta_{ik}) = \sum_{i,j,k\in\{0,1,2\}} \sum_{a,b,c\in\{0,1\}} p_{ia}p_{jb}p_{kc}|i-j||ia-kc| \\ \quad = p_{0\cdot}(p_{11}+2p_{21})(p_{1\cdot}+2p_{2\cdot}) + p_{10}(p_{11}+2p_{21})(p_{0\cdot}+p_{2\cdot}) \\ \quad\quad + p_{11}(1-p_{11})(p_{0\cdot}+p_{2\cdot}) + p_{20}(p_{11}+2p_{21})(p_{1\cdot}+2p_{0\cdot}) \\ \quad\quad + p_{21}\left(p_{11}+2(p_{\cdot 0}+p_{01})\right)(p_{1\cdot}+2p_{0\cdot}) \\ \mathrm{Cov}(G_{ij},\Delta_{ij}) = \mathrm{E}(G_{ij}\Delta_{ij}) - \mathrm{E}(G_{ij})\mathrm{E}(\Delta_{ij}) \\ \mathrm{Cov}(G_{ij},\Delta_{ik}) = \mathrm{E}(G_{ij}\Delta_{ik}) - \mathrm{E}(G_{ij})\mathrm{E}(\Delta_{ij}) \end{cases} \quad \text{(S5)}$$
